# Murine polyomavirus DNA transitions through spatially distinct nuclear replication subdomains during infection

**DOI:** 10.1371/journal.ppat.1008403

**Published:** 2020-03-23

**Authors:** Douglas K. Peters, Robert L. Garcea

**Affiliations:** 1 Department of Molecular, Cellular, and Developmental Biology, University of Colorado Boulder, Boulder, Colorado, United States of America; 2 BioFrontiers Institute, University of Colorado Boulder, Boulder, Colorado, United States of America; Stony Brook University, UNITED STATES

## Abstract

The replication of small DNA viruses requires both host DNA replication and repair factors that are often recruited to subnuclear domains termed viral replication centers (VRCs). Aside from serving as a spatial focus for viral replication, little is known about these dynamic areas in the nucleus. We investigated the organization and function of VRCs during murine polyomavirus (MuPyV) infection using 3D structured illumination microscopy (3D-SIM). We localized MuPyV replication center components, such as the viral large T-antigen (LT) and the cellular replication protein A (RPA), to spatially distinct subdomains within VRCs. We found that viral DNA (vDNA) trafficked sequentially through these subdomains post-synthesis, suggesting their distinct functional roles in vDNA processing. Additionally, we observed disruption of VRC organization and vDNA trafficking during mutant MuPyV infections or inhibition of DNA synthesis. These results reveal a dynamic organization of VRC components that coordinates virus replication.

## Author summary

The *Polyomaviridae* family of small DNA viruses exhibits a diverse host range, and some of its members infect and cause disease in humans. Polyomavirus genome replication occurs in the host nucleus within dynamic subnuclear domains termed viral replication centers. In addition to viral proteins, many cellular proteins localize to these domains and mediate the replication and resolution of circular viral genomes. Polyomavirus replication center composition has been characterized by confocal fluorescence microscopy, but resolution limits have constrained more detailed analyses. We have now used super-resolution fluorescence microscopy (3D-SIM) to analyze the high-resolution organization of murine polyomavirus replication centers during infection. We found that viral replication center components were organized into at least two spatially and functionally distinct subdomains, and that viral DNA sequentially associated with these subdomains post-synthesis. Additionally, we observed defects in replication center organization and viral DNA trafficking during mutant murine polyomavirus infection or after inhibition of DNA synthesis. Our results identify spatially and functionally distinct subdomains of viral replication centers and provide insight into the trafficking of viral genomes within viral replication centers.

## Introduction

Viruses utilize cellular proteins and signaling pathways in order to replicate, often creating new domains in the infected cell [1–9]. These virus-associated domains are a critical component of the host-pathogen interaction, and their study has informed our understanding of viral replication, as well as the cellular processes that are enlisted to enable infection [10]. We investigated one such domain, termed viral replication centers (VRCs), that form in the nucleus during murine polyomavirus (MuPyV) infections [7,8].

The MuPyV genome encodes six proteins, three of which are expressed early in infection (the tumor, or T-antigens). The large (LT), middle (MT), and small (ST) T-antigens are multifunctional and interact with cellular proteins to permit and enhance infection [11–13]. LT localizes to the nucleus and is enriched at VRCs, where it binds the viral origin of replication and functions as a helicase during viral DNA (vDNA) replication [14,15]. MT is a membrane-bound protein that modulates signaling pathways through phosphatidylinositol 3-kinase (PI3K), *src* family kinases, and protein phosphatase (PP2A) [16–18]. ST is soluble and binds PP2A, affecting the phosphorylation state of many cellular proteins, including protein kinase B (AKT) and mitogen activated protein kinase (MAPK) [13,19,20]. Mutant viruses have been used to analyze these protein interactions and have supported roles for ST and MT in genome replication and virus assembly [8,21–23].

Host DNA replication factors carry out bidirectional replication of the viral genome, producing a Cairns intermediate that must be resolved [24]. Host DNA damage response (DDR) proteins aid in resolving these catenated forms into monomeric circular genomes [25–28]. Fluorescence microscopy has been used previously to characterize the composition of polyomavirus (PyV) replication centers, which are minimally defined as subnuclear domains where LT and vDNA co-localize [8,25]. Several host DNA replication factors, such as replication protein A (RPA) and DNA polymerase δ, have been localized to VRCs [7,29,30], as have DNA damage response (DDR) proteins [7,8,25]. An active DDR is required by many DNA viruses, and each virus may manipulate DDR signaling differently to promote infection [9,31]. PyVs ubiquitously activate and utilize the ataxia-telangiectasia mutated (ATM) and ataxia-telangiectasia and Rad3 related (ATR) kinases, which help regulate the DDR and S-phase checkpoint. Inhibiting these kinases results in aberrant vDNA replication products (*e*.*g*., catenated viral genomes or rolling circle replication) and decreased infectious viral output [8,24–27,30,32–34]. An active, phosphorylated form of the ATM kinase (pATM^S1981^) localizes to MuPyV replication centers, indicating that ATM activity is spatially related to viral genome replication and repair [8]. ATM and ATR regulate the activity of many DNA replication and repair proteins [31,35], and it is unknown which phosphorylation events are required at MuPyV replication centers.

Some VRC-associated proteins, such as RPA, participate in both cellular DNA replication and repair processes [36,37]. RPA is a heterotrimer complex (composed of RPA70, RPA32, and RPA14 subunits) that binds and stabilizes single-stranded DNA (ssDNA) during DNA replication and repair. LT binds the RPA70 subunit during PyV replication, which may contribute to efficient RPA loading onto ssDNA that emerges behind the LT helicase [38–40]. In addition to binding ssDNA, RPA also acts as a scaffold for DNA replication and repair factors. Many RPA binding interactions are controlled by phosphorylation of the RPA32 subunit, and specific residues are phosphorylated by DDR-associated kinases in response to DNA damage. Mutational analyses have indicated that phosphorylation of serines 4 and 8 (pRPA32^S4S8^) occurs downstream of other phosphorylation events and is only found in “hyperphosphorylated” RPA32 (containing five or more phosphorylated residues). pRPA32^S4S8^ binds to ssDNA at DNA damage foci, but does not bind undamaged replication forks [36,41–45]. Both replication- and repair-associated RPA activities may be required at MuPyV replication centers.

Although fluorescence microscopy has previously localized cellular and viral proteins to MuPyV replication centers [7,8], the spatial resolution of conventional fluorescence microscopy limits analysis of VRC organization. We have therefore used 3D structured illumination microscopy (3D-SIM) to investigate MuPyV replication centers in greater spatial detail [46]. Using 3D-SIM we found that VRCs could be further resolved into distinct subdomains that were defined by the presence of either LT or RPA32. EdU labeling indicated that vDNA sequentially trafficked through these VRC subdomains. In addition, VRC organization and vDNA relocalization were disrupted during infection by a viral mutant lacking ST expression or after hydroxyurea treatment, suggesting roles for the viral ST protein and active vDNA synthesis in the organization and function of MuPyV VRCs. These results provide a higher resolution model of MuPyV replication during infection that may be applicable to other DNA viruses.

## Results

### MuPyV replication centers are organized into subdomains

**Fig 1:**
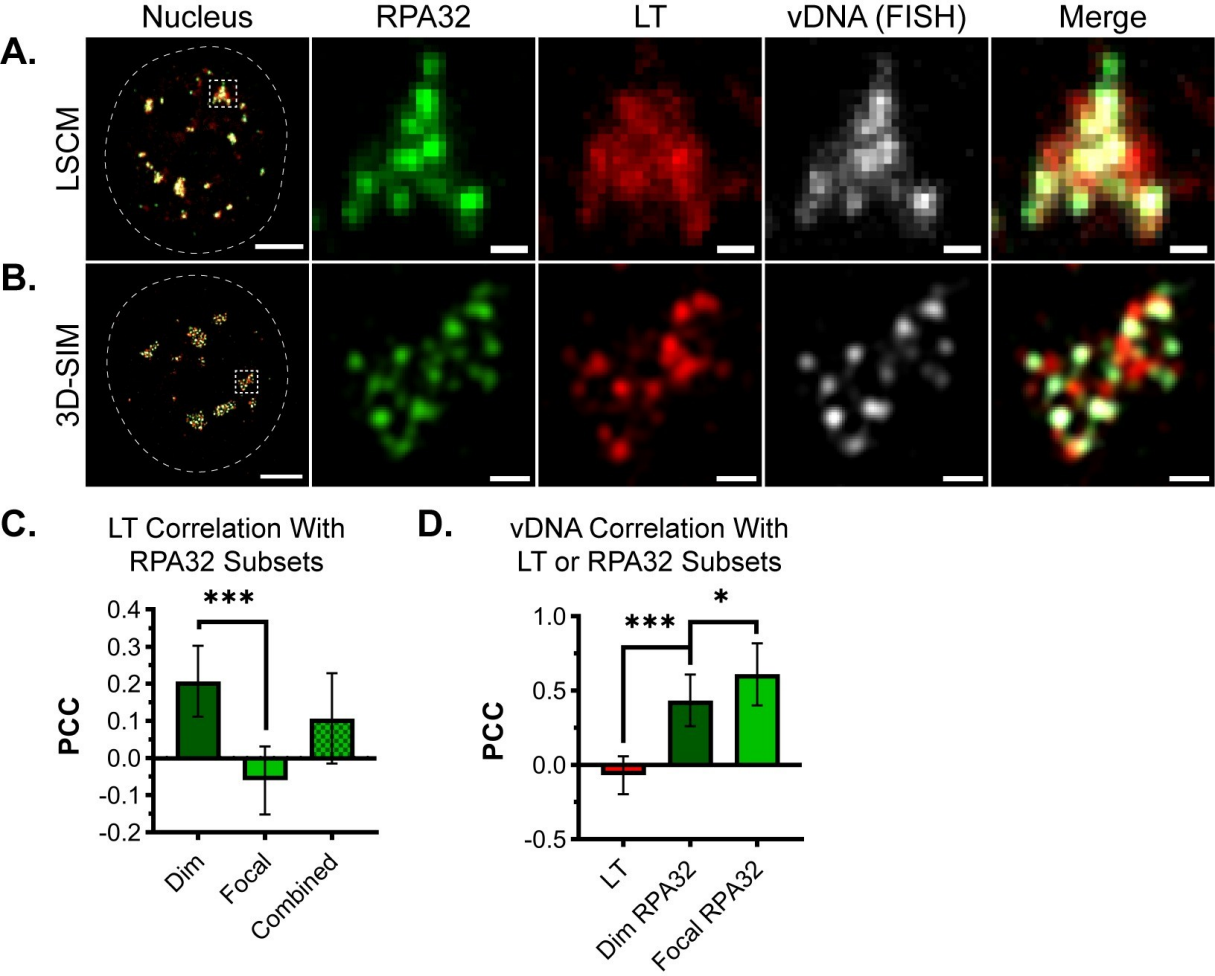
MuPyV replication centers are organized into subdomains. MEFs were infected with WT MuPyV for 30 hrs then fixed and immuno-labeled. Dotted white lines outline the nuclear border, dotted white boxes delineate the cropped viral replication center (VRC) shown in panels to the right of each nucleus. Cells were probed for RPA32 (green), LT (red), and MuPyV DNA (FISH) (gray), then imaged by **either (A)** laser scanning confocal microscopy (LSCM) or **(B)** 3D structured illumination microscopy (3D-SIM). Scale Bars: Nucleus = 5μm, Crop = 0.5μm. **(C)** Pearson’s Correlation Coefficient (PCC) analysis of LT signal with each subset of RPA32 signal (dim, focal, or combined). **(D)** PCC analysis of vDNA signal with either LT, dim, or focal RPA32 signal. Paired t-tests were used to compare mean values (*** = p<0.001; * = p<0.05). n = 13 nuclei.

Proteins involved in cellular DNA replication and repair have been previously localized to VRCs during MuPyV infection, and a subset of these proteins are required for viral genome replication [7,8]. In those studies laser scanning confocal microscopy (LSCM) was used to analyze the co-localization of cellular proteins with LT and vDNA, and the results suggested that these protein components may not be homogenously distributed within VRCs [7,8]. To better understand how host and viral proteins localize within VRCs, we compared images acquired using LSCM (Fig 1A) and 3D-SIM (Fig 1B). MuPyV-infected mouse embryonic fibroblasts (MEFs) were immuno-stained for the previously identified VRC proteins, RPA32 and LT, as well as vDNA using fluorescent *in situ* hybridization (FISH). Although slight differences between RPA32 and LT localization were occasionally resolved using LSCM, 3D-SIM identified distinct VRC subdomains (Fig 1A and 1B, S1A Fig). Each subdomain was defined by either a bright RPA32 or LT fluorescent signal, and line scan analysis suggested that the two signals did not overlap (S1B Fig). These results suggested a level of organization within VRCs that could be resolved further by 3D-SIM.

In addition to bright, focal RPA32, a dim RPA32 subset was observed that overlapped with the localization of LT (Fig 1B). The RPA heterotrimer complex is a critical component of both DNA replication and DNA repair, which are both required for MuPyV infection. Image analysis of the two subsets of RPA32 signal intensity observed by 3D-SIM defined their localization within VRCs. Specifically, the associations of the focal or dim RPA32 subsets with LT were quantified using Pearson’s Correlation Coefficient (PCC). PCCs measure the association of two variables and can be used to calculate the degree of covariance between two fluorescence microscopy images [47]. PCC values range from +1 (perfect correlation) to -1 (perfect anti-correlation). Importantly, PCC analysis of fluorescence images only includes pixels/voxels that co-localize between channels, and it is therefore optimal for measuring changes in functional relationships between two spatially related objects [48].

Isolating the fluorescence signal of interest is a critical step in PCC analysis and was accomplished by thresholding each image to exclude unwanted signal, such as background noise. RPA32 subsets were separated for analysis by setting non-overlapping intensity threshold ranges that specified mutually exclusive regions in each image. These regions represent areas of: (1) unwanted background signal, (2) dim RPA32 signal, and (3) focal RPA32 signal (S1C and S1D Fig). The intensity values delineating each threshold range were determined manually using ImageJ analysis software (see Methods). To validate this approach, PCCs were calculated for LT with dim RPA32, focal RPA32, and both subsets combined (total). LT associated significantly better with dim RPA32 than with focal RPA32 (Fig 1C). Together these results confirmed that the observed distribution of RPA32 represented at least two distinct VRC subdomains: one defined by the presence of LT and dim RPA32, and another by focal RPA32 without LT.

The localization of FISH-labeled vDNA mirrored that of RPA32, in that bright FISH fluorescence overlapped focal RPA32 and dim FISH fluorescence overlapped dim RPA32 (S1A Fig). PCC analysis indicated that the correlation of vDNA with either RPA32 subset (focal or dim) was greater than that of vDNA with LT (Fig 1D). Although LT binds vDNA during its synthesis, these results suggest that the association of vDNA with LT is more transient than the association of vDNA and RPA32.

### Focal RPA32 is associated with DDR signaling proteins

**Fig 2:**
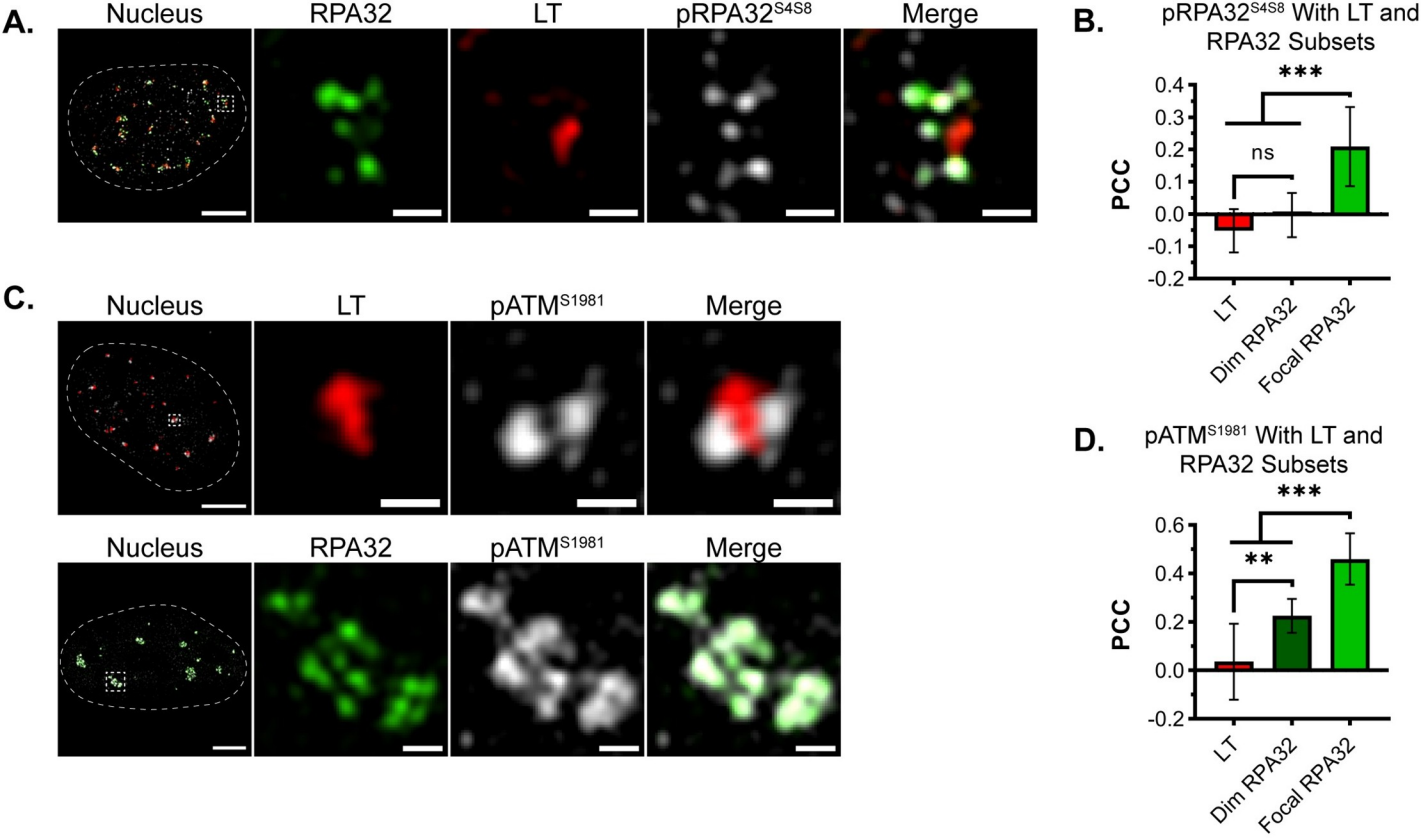
Focal RPA32 is associated with DDR signaling proteins. MEFs were infected with WT MuPyV, processed for microscopy as described in Fig 1, then imaged by 3D-SIM. **(A)** Cells were stained for total RPA32 (green), LT (red), and pRPA32^S4S8^ (gray). pRPA32^S4S8^ associates preferentially with damaged DNA. **(B)** PCC analysis of pRPA32^S4S8^ with either LT, dim RPA32, or focal RPA32. n = 10 nuclei. **(C)** Cells were stained for pATM^S1981^ (gray) and either total RPA32 (green) or LT (red). Protein combinations were labeled and imaged separately due to incompatible antibody sources. **(D)** PCC analysis of pATM^S1981^ with either LT, dim RPA32, or focal RPA32. n = 12 nuclei. Single z-planes of representative nuclei are shown. Scale Bars: Nucleus = 5μm, Crop = 0.5μm. Paired t-tests were used to compare mean values (*** = p<0.001; ** = p<0.01; ns = p>0.05).

In cells undergoing DNA damage repair, RPA forms foci that are similar to those observed in VRCs [49,50]. To determine if the focal RPA32 was associated with DDR signaling, the hyperphosphorylated form of RPA32 (pRPA32^S4S8^) and phosphorylated ATM kinase (pATM^S1981^) were visualized. Most of the pRPA32^S4S8^ overlapped the focal RPA32 signal, suggesting these sites were associated with “damaged” vDNA (Fig 2A and S2A Fig). PCC analysis quantified the relative strengths of pRPA32^S4S8^ association with LT, dim RPA32, or focal RPA32 within several nuclei. The correlation of pRPA32^S4S8^ with focal RPA32 was significantly greater than with dim RPA32 or LT (Fig 2B). This analysis identified a subpopulation of RPA32 in the VRCs that is hyperphosphorylated and associated with DDR signaling.

The localization of pATM^S1981^ was similar to what was observed for pRPA32^S4S8^ (Fig 2C and S2B Fig), further indicating that focal RPA32 was associated with DDR signaling. PCC analysis indicated the correlation of pATM^S1981^ with RPA32 (focal or dim) was significantly greater than with LT. Notably, the correlation of pATM^S1981^ with focal RPA32 was significantly greater than with LT, suggesting pATM^S1981^ was associated with RPA-coated vDNA (Fig 2D). These results suggest the co-localization of pRPA32^S4S8^, pATM^S1981^, and focal RPA32 within a VRC subdomain that may be associated with vDNA processing steps.

### Nascent MuPyV DNA rapidly dissociates from LT

**Fig 3:**
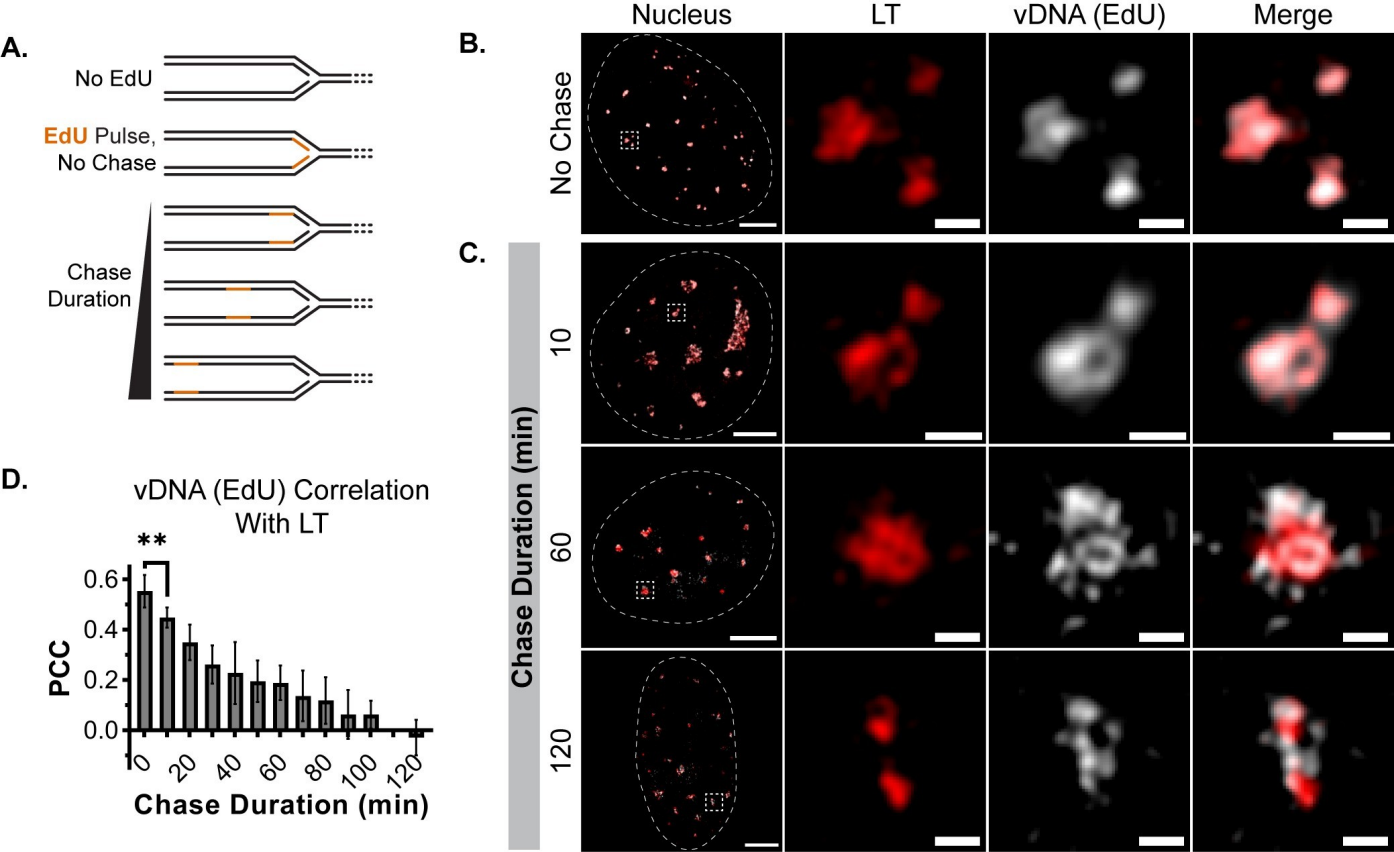
Nascent MuPyV DNA rapidly dissociates from LT. MEFs were infected with WT MuPyV and incubated in the presence of EdU for 5 min, then either fixed immediately (No Chase) or incubated in EdU-free media for the indicated chase duration prior to being fixed. The infection was allowed to proceed until 30 HPI, at which point all conditions were fixed and stained for LT (red) and EdU (gray), then imaged by 3D-SIM. **(A)** Schematic representation of EdU pulse-chase experiment indicating the position of EdU-labeled DNA (orange) relative to a replication fork as a function of chase duration. **(B-C)** Single z-planes of infected nuclei that were pulsed with EdU and either fixed (No Chase) or chased without EdU for 10, 60, or 120 min prior to fixation. Dotted white lines outline the nuclear border, dotted white boxes delineate the cropped VRC shown in panels to the right of each nucleus. Scale Bars: Nucleus = 5μm, Crop = 0.5μm. **(D)** PCC analysis of vDNA (EdU) with LT. Unpaired t-test was used to compare mean values (** = p<0.01). n = 6 nuclei per time point.

Although FISH identified vDNA at DDR-associated focal RPA32 (Figs 1 and 2), the trafficking of vDNA between VRC subdomains was unclear. EdU labels replicating vDNA within PyV replication centers [8,51,52], so we applied pulse-chase labeling with EdU to identify discrete subpopulations of vDNA at different times post-synthesis (Fig 3A). The EdU signal immediately after the pulse label (nascent vDNA) localized within VRCs and overlapped with LT, suggesting this subdomain represented the initial site of vDNA synthesis (Fig 3B and S3A Fig). The EdU and LT signals overlapped less as the chase duration increased to 120 min, reflecting a progressive spatial separation of vDNA and LT (Fig 3C and S3B Fig).

PCC analysis quantified the association of EdU and LT during the pulse-chase (Fig 3D). The average PCC value of EdU-labeled vDNA with LT was highest immediately after the pulse-label and decreased by nearly 20% between 0 and 10 min post-synthesis (0.553 to 0.447, p<0.01), indicating the rapidity with which LT and vDNA began to dissociate. The average PCC values decreased steadily over the two hour chase (0.553 to -0.030 by 120 min). vDNA appeared to accumulate adjacent to LT after dissociating, suggesting that additional vDNA processing steps occur within VRCs.

### MuPyV DNA progresses from LT to focal RPA32 subdomains

**Fig 4:**
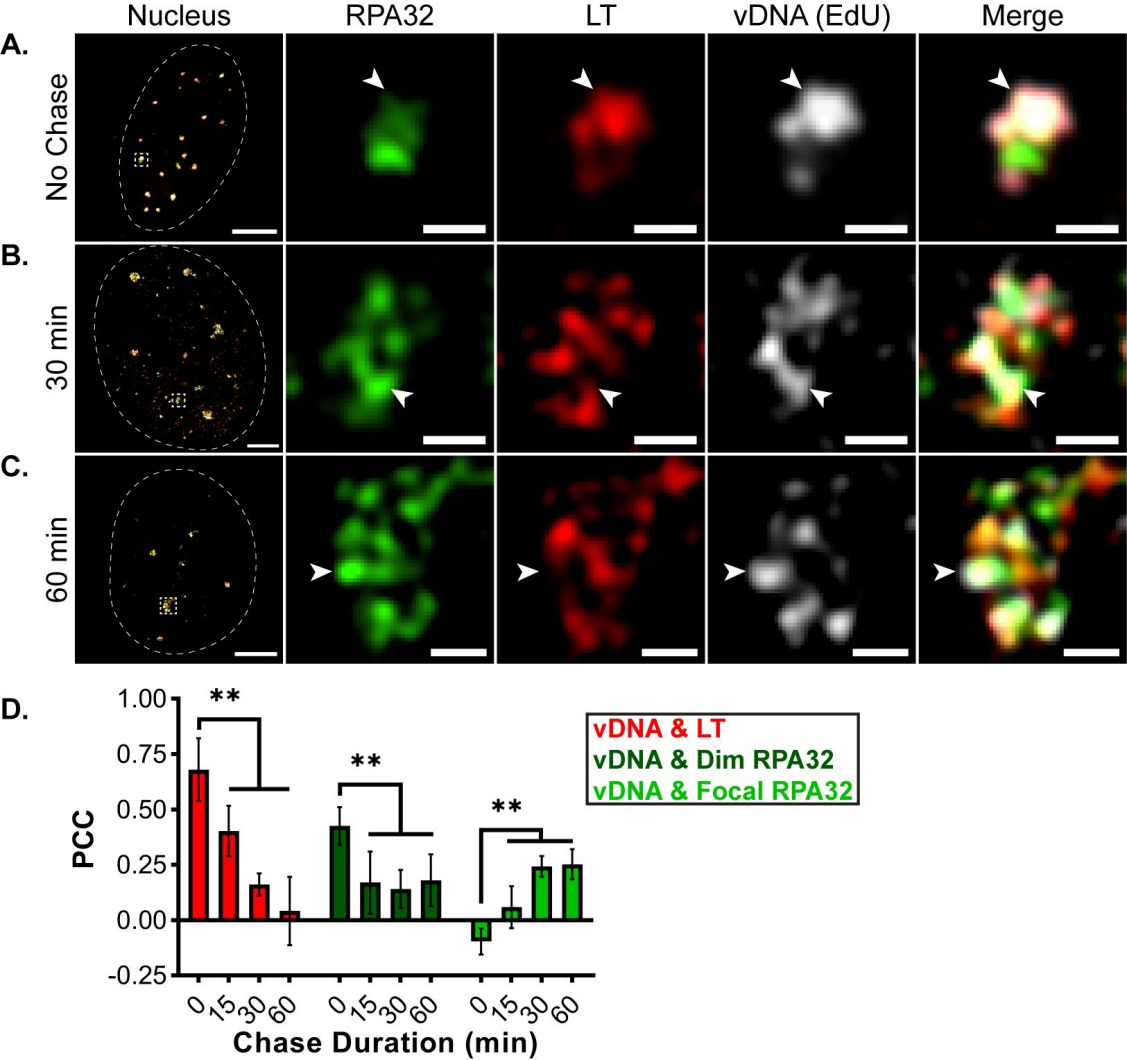
MuPyV DNA progresses from LT to focal RPA32 subdomains. MEFs were infected with WT MuPyV and processed and labeled as described in Fig 3. Cells were stained for RPA32 (green), LT (red), and EdU-labeled vDNA (gray), then imaged by 3D-SIM. A single z-plane of representative nuclei from: **(A)** 0 min (no chase), **(B)** 30 min, and **(C)** and 60 min chase conditions. Arrowheads in each panel indicate regions of vDNA (EdU) localization relative to RPA32 and LT. Scale Bars: Nucleus = 5μm, Crop = 0.5μm. **(D)** PCC analysis of vDNA (EdU) with either LT, dim RPA32, or focal RPA32. Unpaired t-tests were used to compare mean values (** = p<0.01). n = 6 nuclei per time point.

Because the 3D-SIM results suggested that MuPyV replication centers could be resolved into at least two spatially distinct subdomains defined by LT and RPA32 (Fig 1), we determined if post-synthesis vDNA relocalized to focal RPA32. As shown above, nascent EdU-labeled vDNA overlapped LT immunofluorescence within VRCs (Fig 4A, arrowhead). However, after a 30 min chase, EdU-labeled vDNA overlapped both LT and focal RPA32 (Fig 4B, arrowheads), and by 60 min vDNA almost completely overlapped focal RPA32, with very little signal remaining associated with LT (Fig 4C, arrowhead). Line scan analysis verified this shift in localization (S4A Fig), and PCC analysis corroborated the visual observations (Fig 4D). Specifically, the average PCC of vDNA with LT decreased significantly over the first 60 min post-synthesis (Fig 4D, red), and a similar trend was observed in the PCC values of vDNA with dim RPA32 (Fig 4D, dark green). Finally, the correlation of vDNA with focal RPA32 increased significantly over the same time frame (Fig 4D, bright green). Together, these results show that vDNA relocalized from LT to focal RPA32 within the first hour after vDNA synthesis.

### ST contributes to vDNA relocalization and VRC organization

**Fig 5:**
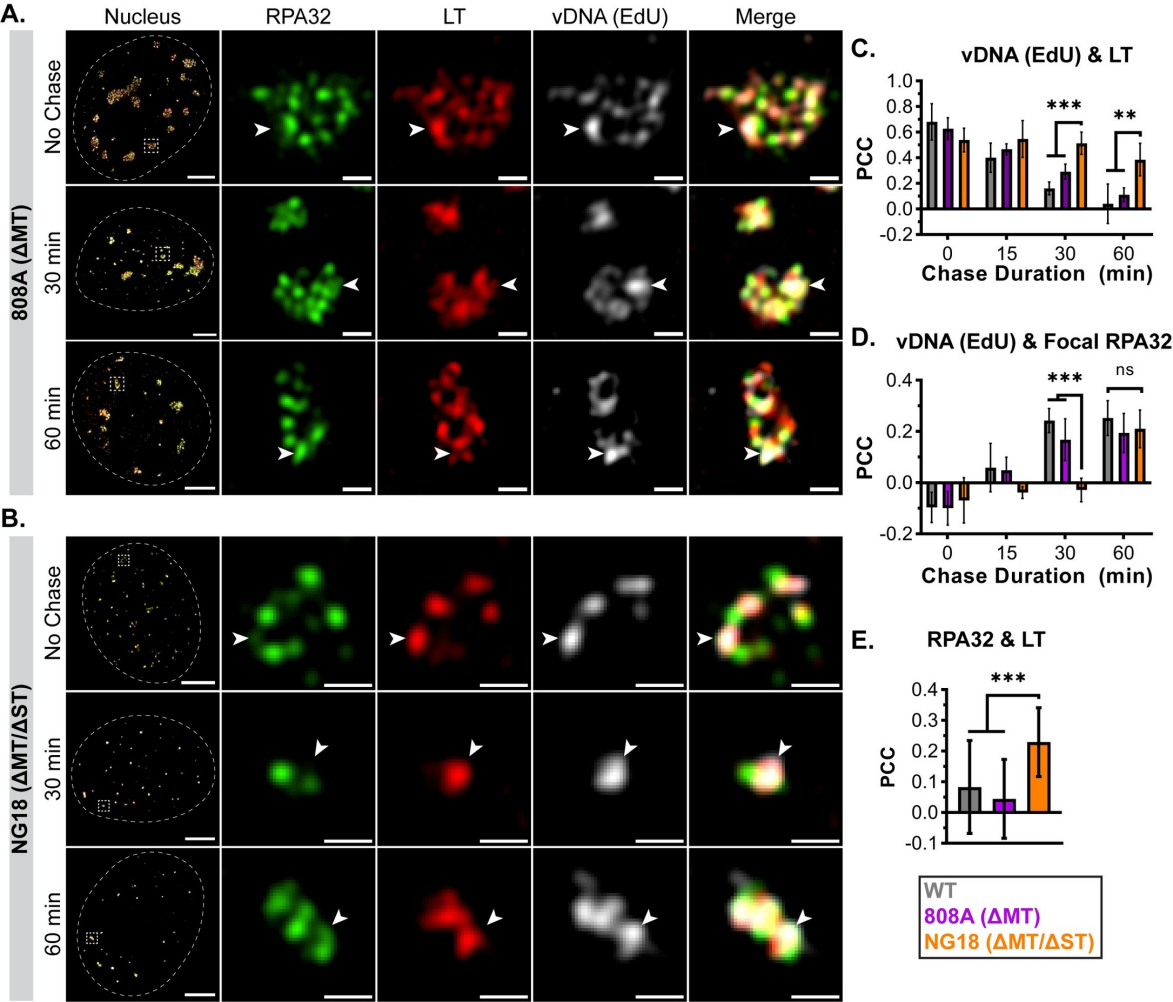
ST contributes to vDNA relocalization after synthesis. MEFs were infected with mutant MuPyVs 808A (ΔMT) or NG18 (ΔMT/ΔST), then processed and labeled as described in Fig 3. Cells were stained for RPA32 (green), LT (red), and EdU (gray), then imaged by 3D-SIM. A single z-plane of representative nuclei from 0 min (No Chase), 30 min, and 60 min chase conditions in **(A)** 808A-infected or **(B)** NG18-infected cells. Arrowheads in each panel indicate regions of vDNA (EdU) localization relative to RPA32 and LT. Scale Bars: Nucleus = 5μm, Crop = 0.5μm. PCC values calculated for vDNA (EdU) with either **(C)** LT or **(D)** focal RPA32, and were plotted alongside PCC values from WT-infected cells (Fig 4). n≥5 nuclei per time point. **(E)** PCC values for combined RPA32 and LT, pooled across time points. n≥23 nuclei per virus. Unpaired t-tests were used to compare mean values (*** = p<0.001; ** = p<0.01; ns = not significant).

The 808A virus lacks MT expression and accumulates vDNA to similar levels as WT (NG59RA) virus during infection, but exhibits an encapsidation/assembly defect [22,23]. The additional loss of ST expression in the mutant viruses NG18 and NG59 results in both reduced vDNA accumulation and production of infectious viral particles, suggesting that ST functions to facilitate vDNA replication [21,22]. Consistent with the reduction of viral replication and assembly functions, NG18 replication centers are smaller than those formed during WT or 808A infections [8]. Therefore, we hypothesized that defects in NG18 replication may affect VRC subdomain organization. Cells infected with WT, 808A, or NG18 were immuno-labeled for RPA32 and LT after an EdU pulse-chase. Similar to WT, RPA32 and LT were localized into spatially distinct VRC subdomains during 808A or NG18 infections (Fig 5A and 5B, respectively), and line scan analysis verified this observation (S5A and S5B Fig). Consistent with our previous report, a distribution of VRC sizes were detected in WT- and 808A-infected cells, whereas large NG18 VRCs were never observed [8]. These results suggested that segregation of RPA32 and LT into subdomains was not dependent on MT or ST, but that VRC “expansion” required ST.

PCC analysis quantified the associations of RPA32, LT, and vDNA at each time point after synthesis. In 808A-infected cells, there were no significant differences from WT in the kinetics of vDNA relocalization from LT to focal RPA32 (Fig 5C–5E, violet). In NG18-infected cells, however, the association of vDNA and LT was significantly prolonged (Fig 5C, orange). Furthermore, the increase in the correlation of vDNA with focal RPA32 was delayed relative to WT (Fig 5D, orange). These results show that neither ST nor MT are required for the segregation of the RPA32 and LT into subdomains within VRCs, but that ST facilitates the relocalization of vDNA from LT to focal RPA32.

One possible explanation for these observations is that vDNA synthesis proceeds more slowly in NG18 VRCs than in WT VRCs. Because RPA levels have been observed to increase at stressed replication forks in order to stabilize the extended stretches of ssDNA and initiate the DDR [53–56], we analyzed RPA32 localization at sites of NG18 vDNA synthesis. PCC analysis showed that the correlation of LT with dim RPA32 was significantly higher in NG18-infected cells than in WT-infected cells (Figs 5E and S5C, orange), possibly representing increased RPA-bound ssDNA and replication stress [53–56].

**Fig 6:**
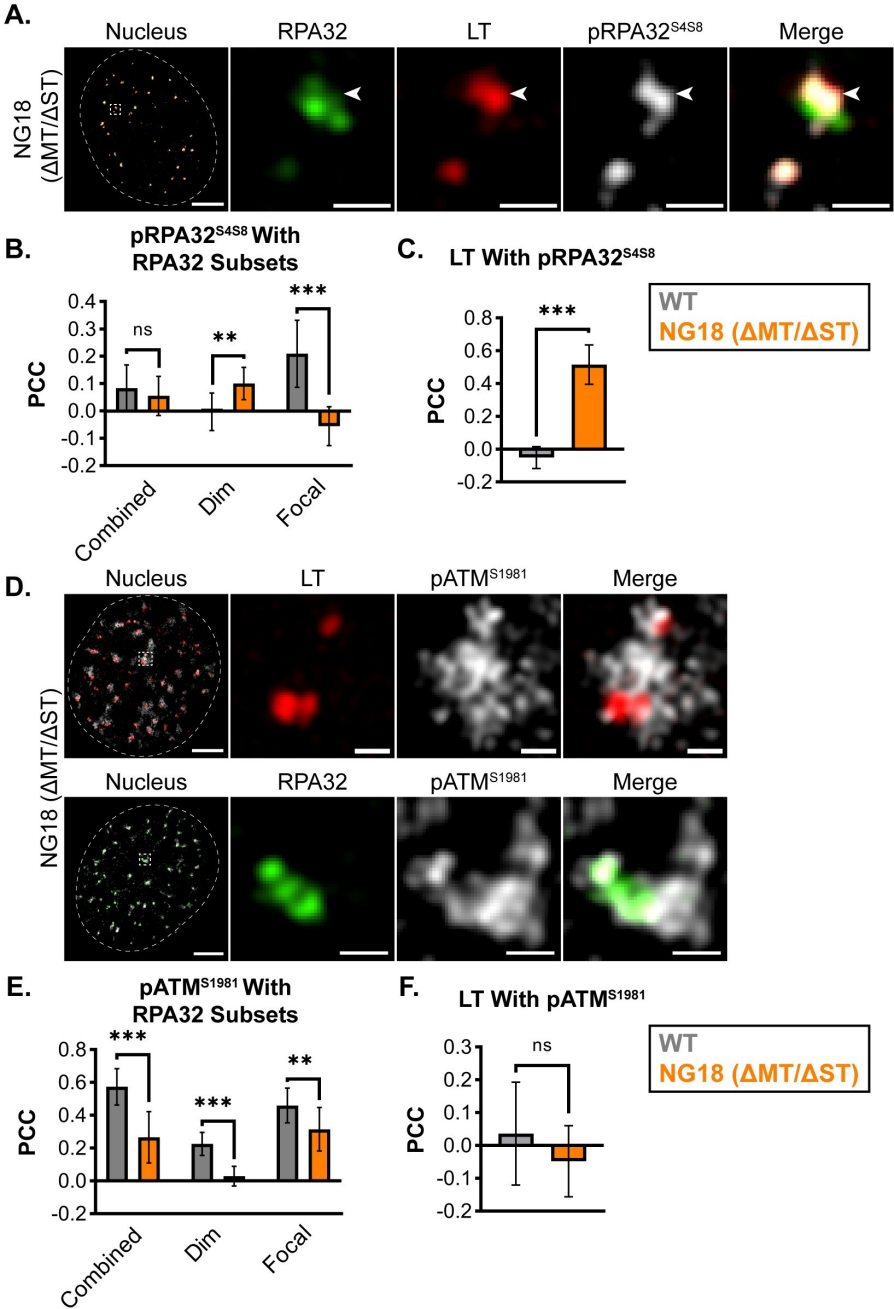
pRPA32^S4S8^ and pATM^S1981^ localization is disrupted in NG18 VRCs. MEFs were infected with NG18 (ΔMT/ΔST), then processed and labeled as described in Fig 2, then imaged by 3D-SIM. **(A)** Cells were stained for RPA32 (green), LT (red), and pRPA32^S4S8^ (gray). Arrowhead indicates region of pRPA32^S4S8^ localization relative to RPA32 and LT. **(B)** PCC analysis of the pRPA32^S4S8^ signal with combined, dim, or focal RPA32 subsets in WT- and NG18-infected cells. **(C)** PCC analysis of LT with pRPA32^S4S8^. n = 10 nuclei. **(D)** Cells were stained for pATM^S1981^ (gray) and either total RPA32 (green) or LT (red). Protein combinations were labeled and imaged separately due to incompatible antibody sources. **(E)** PCC analysis of pATM^S1981^ with combined, dim, or focal RPA32 subsets in WT- and NG18-infected cells. **(F)** PCC analysis of LT with pATM^S1981^. n = 12 nuclei. Single z-planes of representative nuclei are shown. Scale Bars: Nucleus = 5μm, Crop = 0.5μm. Unpaired t-tests were used to compare mean values (*** = p<0.001; ** = p<0.01; ns = p>0.05).

To determine if NG18 VRCs exhibited other markers of replication stress, we compared the localization of pRPA32^S4S8^ to that found in WT-infected cells (Fig 2A and 2B). We found that instead of overlapping with focal RPA32, pRPA32^S4S8^ in NG18-infected cells overlapped with LT (Fig 6A and S6A Fig). PCC analysis confirmed a significantly greater association of pRPA32^S4S8^ with LT or dim RPA32 in NG18-infected cells, and the correlation of pRPA32^S4S8^ with focal RPA32 was significantly reduced in NG18-infected cells (Fig 6B and 6C). These results suggested that sites of NG18 vDNA synthesis exhibit markers of both ssDNA accumulation and replication stress.

To determine if other VRC components may be disrupted during NG18 infection, we visualized pATM^S1981^ localization in NG18-infected cells. In WT-infected cells, pATM^S1981^ correlated well with focal RPA32 (Fig 2C and 2D), but its localization changed significantly in NG18-infected cells. In addition to diminished overlap with focal RPA32, we also observed VRC-associated pATM^S1981^ signal that did not overlap RPA32 or LT at all (Fig 6D and S6D Fig). PCC analysis confirmed that the correlation of pATM^S1981^ signal significantly decreased with dim RPA32 and with focal RPA32 (Fig 6E), and that pATM^S1981^ did not relocalize to LT (Fig 6F), as occurred with pRPA32^S4S8^ localization. These results suggested that VRC components exhibit significant differences in localization during NG18 infection, and that ST contributes to the organization of VRC components observed in WT-infected cells.

### Hydroxyurea reversibly disrupts VRC organization

**Fig 7:**
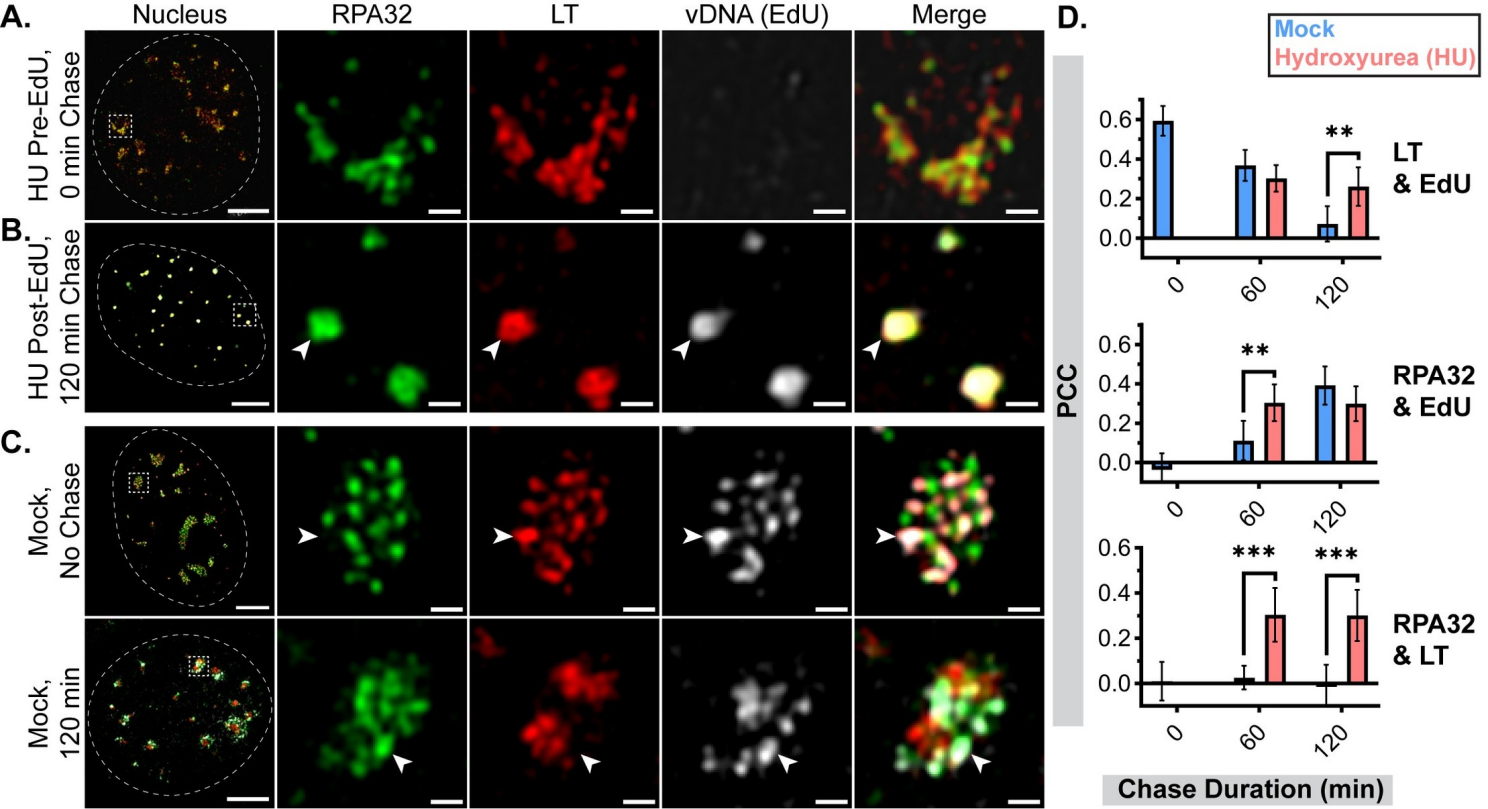
Hydroxyurea disrupts VRC organization and vDNA relocalization. MEFs were infected with WT MuPyV, then processed and labeled as described in Fig 4. Chase media also contained 5mM hydroxyurea (HU) in indicated conditions. Cells were stained for RPA32 (green), LT (red), and EdU (gray), then imaged by 3D-SIM. A single z-plane of representative image from: **(A)** Cells treated with HU for 60 min *prior* to EdU pulse, **(B)** Cells treated with HU for 120 min after the EdU pulse, and **(C)** Cells chased into HU-free media after EdU pulse for 0 min (No Chase, top) or 120 min (bottom). Arrowheads in each panel indicate regions of vDNA (EdU) localization relative to LT and RPA32. Scale Bars: Nucleus = 5μm, Crop = 0.5μm. **(D)** PCC analysis of each pair of fluorescent channels (>99.75% threshold for all): LT with EdU (top), RPA32 with EdU (middle), RPA32 with LT (bottom). Unpaired t-tests were used to compare mean values (*** = p<0.001, ** = p<0.01). n≥6 nuclei per condition.

Hydroxyurea (HU) was used to determine if active vDNA replication contributes to VRC organization [54,57–59]. RPA32, LT, and EdU-labeled vDNA were localized in MuPyV-infected cells treated with HU. No EdU signal was detected in cells treated with HU for 1 hr prior to an EdU pulse, confirming inhibition of DNA synthesis (Fig 7A). HU addition disrupted the formation of distinct RPA32 and LT subdomains, instead creating foci that included both RPA32 and LT (Fig 7B and S7A and S7B Fig). In addition, the vDNA remained in these foci with both LT and RPA32 (Fig 7C and S7C Fig). PCC analysis confirmed these observations, indicating greater correlations in HU-treated cells for each pair of fluorescent labels (Fig 7D). Thus, HU affected the formation of distinct domains for RPA32 and LT by blocking vDNA synthesis and its subsequent relocalization.

**Fig 8:**
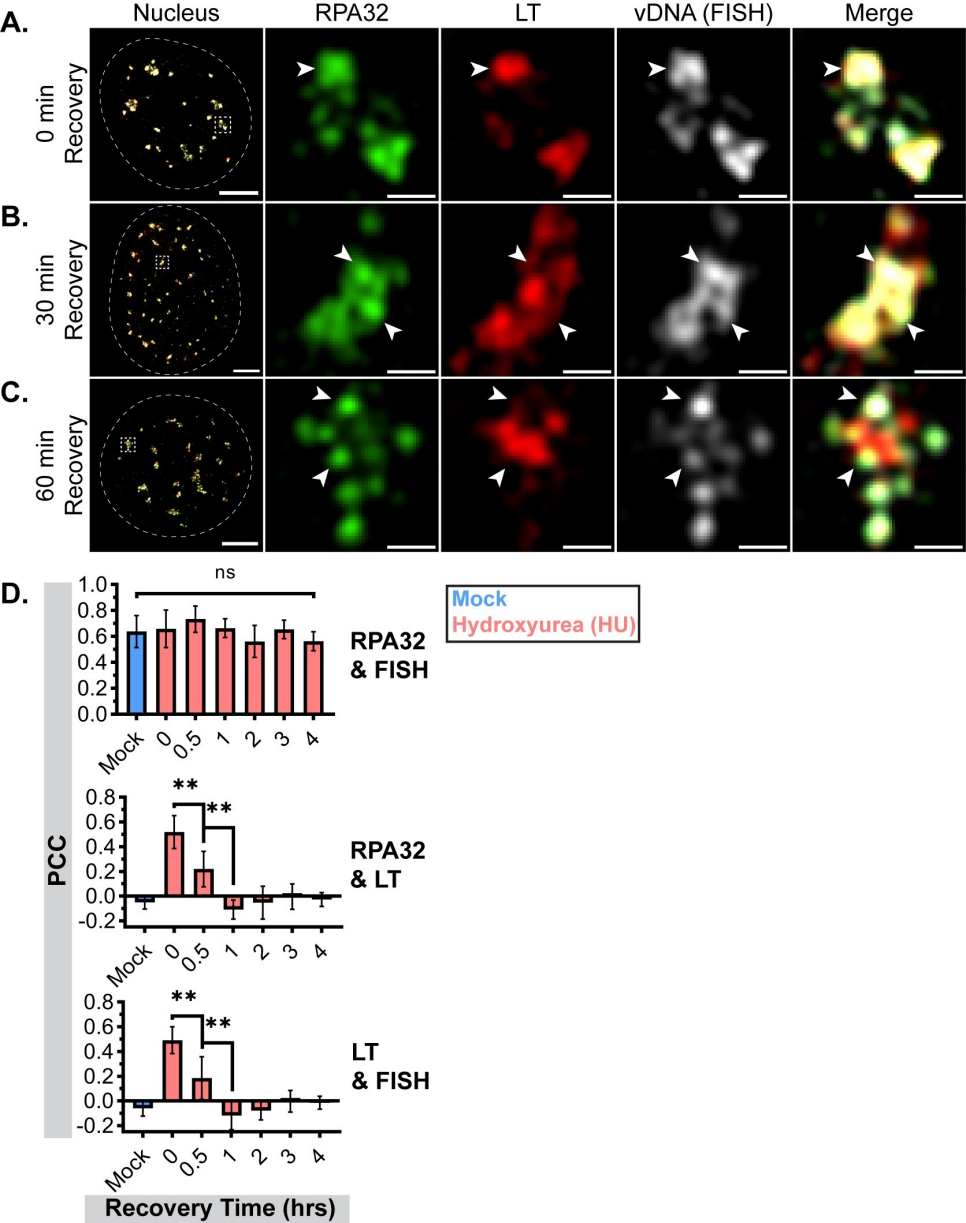
VRC disruption by hydroxyurea is reversible. MEFs were infected with WT MuPyV and treated with HU for 30 min at different times prior to fixation. Cells were then incubated in HU-free media until 32 HPI, at which point all conditions were fixed and processed as described in Fig 4. Cells were stained for RPA32 (green), LT (red), and vDNA (gray) using fluorescent *in situ* hybridization (FISH) probes against the viral genome. A single z-plane is shown from: **(A)** Cells fixed immediately after 30 min HU pulse, **(B)** Cells allowed to recover in HU-free media for 30 min, and **(C)** Cells allowed to recover in HU-free media for 60 min. Arrowheads in each panel indicate regions of vDNA (FISH) localization relative to LT and RPA32 signal. Scale Bars: Nucleus = 5μm, Crop = 0.5μm. **(D)** PCC analysis of each pair of fluorescent channels (>99.75% threshold for all): RPA32 with vDNA (top), RPA32 with LT (middle), LT with vDNA (bottom). Unpaired t-tests were used to compare mean values (** = p<0.01, ns = not significant). n = 6 nuclei per condition.

We next determined whether VRC organization could be restored after HU removal. MuPyV-infected cells were treated with HU for 30 min, then changed to drug-free media and allowed to recover for up to 4 hrs. Similar to longer treatments (Fig 7), the overlap of RPA32 and LT was also increased after a 30 min HU treatment (Fig 8A and S8A Fig). In addition, FISH labeling showed that vDNA was predominantly localized within HU-disrupted VRCs (Fig 8B and S8B Fig). Within the first hour after removing HU, RPA32 and LT relocalized to the subdomains seen in mock-treated cells (Fig 8C and S8C Fig), and PCC analysis supported these observations (Fig 8D). Relative to mock-treated cells, the correlation of LT with RPA32 (or vDNA) increased significantly immediately after the 30 min HU treatment, subsequently decreased by 40% by 30 min after removing HU, and then became indistinguishable from mock-treated cells by 60 min after removing HU. Together these results show that inhibition of vDNA synthesis by HU caused a rapid, albeit reversible, disruption of VRC organization that prevented vDNA trafficking from LT to RPA32 subdomains.

## Discussion

Using 3D-SIM we observed that MuPyV nuclear replication centers are organized into at least two spatially and functionally distinct subdomains associated with either vDNA replication or repair. A “replication-associated” subdomain was defined by bright LT and dim RPA32 signal (Fig 1), as well as nascent EdU-labeled vDNA (Figs 3 and 4). A “repair-associated” subdomain was defined by focal RPA32, pRPA32^S4S8^, and pATM^S1981^ localization (Figs 1 and 2), and the accumulation of vDNA (Figs 1 & 4). During these time frames we did not detect a subsequent decrease in the association of focal RPA32 and EdU-labeled vDNA that might represent the transition of vDNA to another site, such as one associated with transcription or virus assembly. Longer chase times after EdU labeling may detect the next phases of vDNA localization, further defining the pathways of vDNA trafficking after synthesis. MuPyV vDNA may accumulate within other nuclear domains, such as promyelocytic nuclear bodies (PML-NBs), which house proteins involved in DNA metabolism and the innate immune response [60]. The formation and functions of PML-NBs are often affected by DNA viruses during infection, including PyVs [61–64]. Notably, a previous study of two PyVs (SV40 and BKV) used a BrdU pulse-chase and confocal fluorescence microscopy to visualize the relocalization of a subset of vDNA from replication centers to adjacent PML-NBs several hours after synthesis [65]. Although PML-NBs also localize near VRCs in MuPyV-infected cells, the PML protein itself is not required for MuPyV infection *in vitro* or *in vivo* [7]. Furthermore, we did not observe regions of EdU- or FISH-labeled vDNA that would be consistent with the size or morphology of PML-NBs (Figs 1 and 3–5). Further research is required to determine the interaction of PML-NBs and PyV infection. Nonetheless, our current results indicate that, in the first two hours after its synthesis, MuPyV DNA dynamically associates with functional subdomains within MuPyV replication centers.

We extended the analysis by studying VRC organization during infection with MuPyV mutant viruses. 808A and NG18 VRCs exhibited a segregation of LT and focal RPA32 similar to that observed in WT VRCs, but during NG18 infection there was a delay in vDNA relocalization from LT to focal RPA32 (Fig 5). NG18-infected cells also exhibited markers of possible replication stress, such as increased localization of both RPA32 and pRPA32^S4S8^ to sites of vDNA synthesis (Fig 6) [57,58]. These results suggest that ST facilitates efficient vDNA replication, as evidenced by vDNA translocation from the replication-associated subdomain (LT) to the repair-associated subdomain (focal RPA32). The localization of pATM^S1981^ was also disrupted in NG18-infected cells. The correlation of pATM^S1981^ with RPA32 (both dim and focal) significantly decreased in NG18-infected cells, and there were regions of pATM^S1981^ signal in VRCs that were in association with neither RPA32 nor LT (Fig 6). These results suggest that ST is required for the organization of VRC components observed in WT-infected cells. The protein partners mediating the ST effects are unclear, but its major binding partner, PP2A, is a likely candidate. Previous studies of polyomavirus ST/PP2A complexes have focused on cell proliferation and cell cycle progression [66–68], but PP2A also regulates DNA replication and repair directly [69–71]. PP2A modulation by ST may alter the localization and activity of VRC-associated proteins, including RPA32/pRPA32^S4S8^ and pATM^S1981^ [71,72]. Additional analysis of VRC composition and organization by super-resolution microscopy may further our understanding of how ST/PP2A complexes enhance vDNA replication.

We found that HU disrupted VRC organization and vDNA trafficking, relocalizing RPA32 and LT into a single subdomain with the vDNA (Fig 7). These observations suggest that HU stalled vDNA replication forks, where the RPA complex would be required to stabilize ssDNA and where nascent vDNA would be sequestered in the absence of ongoing replication. The reversibility of this disruption suggests that HU only temporarily stalls vDNA replication forks without causing their irreparable collapse (Fig 8). The mechanism of its reversal is unknown, but these results suggest that active vDNA replication contributes to VRC organization.

Our analysis focused on the localization of viral LT and cellular RPA32, which are involved in the early steps of viral genome replication that could be analyzed with EdU and FISH labeling. Although vDNA was initially associated with LT and RPA32, these proteins represent only a small subset of proteins involved in vDNA replication and repair. Additional proteins have been previously localized to MuPyV replication centers by conventional fluorescence microscopy, including pCHK1^S345^, MRE11a, pATM^S1981^, and γH2AX [8]. Studies of the 3D-SIM localization of these proteins (and others) during vDNA labeling may further define the composition and functions of VRC subdomains. For example, super-resolution fluorescence microscopy was recently used to analyze the transition of damaged DNA from globular RPA clusters to RAD51 filaments during homologous recombination repair [50,73]. Similar analyses of MuPyV replication center components will help define the spatial and temporal characteristics of vDNA replication and repair pathways during infection.

Previous immuno-electron microscopy of JCV-infected SVG-A cells described a close spatial relationship between VRCs and assembled virions [51], and a similar relationship was observed during MuPyV infection [7]. The spatial coordination of these processes presumably enhances infection by accumulating and maintaining cellular and viral factors adjacent to sites of vDNA synthesis. Many cellular proteins are likely required for each of these processes, and targeted approaches (such as fluorescence microscopy) are unlikely to identify all the relevant proteins. Isolation of proteins on nascent DNA (iPOND) may provide a complementary biochemical approach. The iPOND technique combines EdU labeling with mass spectrometry to identify proteins associated with EdU-labeled DNA, and was used previously to identify proteins involved in herpes simplex virus (HSV) and adenovirus replication [74–76]. iPOND may identify new MuPyV replication center protein candidates, and 3D-SIM localization can be used to validate these candidates, localizing them within VRC subdomains in relation to other replication factors.

The segregation of VRC components into subdomains likely has important functional consequences for viral genome replication, but the mechanisms underlying subdomain formation are unclear. One possibility is that subdomains form by a DNA-mediated compartmentalization mechanism similar to that proposed for HSV, where the abundance of accessible (*i*.*e*., chromatin-free) vDNA binding partners leads to the enrichment of DNA-binding proteins at VRCs [77]. Regions within VRCs where distinct vDNA subpopulations accumulate may each exhibit a different set of DNA-protein interactions, resulting in the segregation of the functionally and spatially distinct subdomains observed by 3D-SIM. Additionally, these subdomains may keep proteins with opposing functions spatially separated during vDNA replication and repair. Such a model may explain how vDNA replication continues during robust DDR and S-phase checkpoint activation, which typically inhibits replication machinery until the damage is resolved [8,76,78].

Many DNA viruses, including adenoviruses, papillomaviruses, herpesviruses, and polyomaviruses, form nuclear replication compartments during infection [1,9]. Fluorescence microscopy has recently revealed complexities in the organization and function of these compartments. Human cytomegalovirus DNA was shown to be synthesized on the replication compartment periphery and subsequently relocalize to its interior [79], and adenovirus DNA undergoes multiple shifts in localization and protein associations throughout infection [80–82]. Super-resolution microscopy may clarify the composition, organization, and functionality of these complex host-pathogen interfaces, especially in replication compartments where diffraction-limited microscopy is insufficient to resolve internal characteristics. Herpes simplex virus 1 offers an example where super-resolution microscopy revealed replication compartment “sub-structures,” in which replication and transcription occur separately [83]. Our results suggest super-resolution microscopy also enables the study of MuPyV replication center subdomains. These examples all use different proteins and utilize different cellular signaling pathways than MuPyV, but we hypothesize that viral replication center organization and function may be fundamentally related between virus families.

## Methods

### Cell lines and virus infections

C57BL/6 mouse embryonic fibroblasts (MEFs) were obtained from ATCC (SCRC-1008; Manassas, VA). MEFs were grown in DMEM (D6429, Sigma) supplemented with 10% fetal bovine serum (FBS; F0926, Sigma), 1x Antibiotic-Antimycotic (A-A, Gibco), 55μM β-mercaptoethanol (βME) at 37°C with 5% CO_2_. Virus strain NG59RA was used for all WT virus infections [84]. Virus strain NG18 has a deletion that abrogates MT and ST expression [21,22]. Virus strain 808A has a mutation in the MT splice acceptor that only prevents the expression of MT (LT and ST expression is unaffected) [22,23]. For infections, MEFs were grown to 40% confluency and then cultured overnight in DMEM / A-A / βME without serum to increase the proportion of infectible cells. Virus was diluted in adsorption buffer (Hanks Balanced Salt Solution (HBSS) / 10mM HEPES, pH 5.6 / 0.5% bovine calf serum (BCS)) and added to cells as previously described [85] to yield a 50% infection efficiency. Cells were incubated in the presence of virus for 2 hrs at 37°C and 5% CO_2_, after which the virus supernatant was removed and replaced with post-infection media (DMEM / 1% FBS / A-A / βME) for the remainder of the experiment. Hydroxyurea (HU, Sigma H8627) was dissolved in DMEM / 1% FBS / A-A / βME and added to cells at indicated times post-infection.

### Immunofluorescence

MEFs were cultured on acid-etched glass coverslips (12mm, No. 1.5) and infected as described above. Cells were pre-extracted and fixed as described previously [8]. Following an overnight block in 10% BCS in PBS (block solution) at 4°C, cells were incubated with primary antibody diluted in block solution at 37°C for 2 hrs, rinsed twice and incubated in block solution for 30 min at RT. For pATM^S1981^ immunolabeling only, cells were instead incubated with primary antibody diluted in block solution overnight at 4°C. Cells were then incubated for 2 hrs at 37°C with AlexaFluor-conjugated secondary antibodies diluted in block solution. Finally, cells were washed three times with PBS and mounted onto slides with ProLong Glass Antifade Mountant (P36980, Invitrogen) and allowed to cure at RT for at least two days before imaging.

### Immunofluorescence antibodies

Primary antibodies used for immunostaining were: mouse anti-TAg (Ab-4, 1:100, Calbiochem) [86]; rat anti-TAg (E1, 1:4000, gift from Tom Benjamin); rat anti-RPA32 (4E4, 1:10, gift from Heinz-Peter Nasheuer) [87]; rabbit anti-pRPA32^S4S8^ (ab87277, 1:200, AbCam); mouse anti-pATM^S1981^ (clone 10H11.E12, 1:250, Millipore Sigma). Primary antibodies were detected using secondary antibodies conjugated with AlexaFluor 488, AlexaFluor 546, or AlexaFluor 647 (Invitrogen), and diluted to 1:1500. All primary and secondary antibodies were diluted in 10% BCS in PBS.

### Fluorescent in Situ Hybridization (FISH)

FISH probes against the MuPyV genome (NG59RA) were generated and analysis was performed as described previously [8]. Briefly, cells were grown on coverslips, infected, fixed, and immuno-labeled as described above, then fixed again in 3% PFA for 10 min to cross-link bound antibodies. Cells were rinsed once with 2x SSC, followed by RNase treatment. The FISH probe was diluted 1:50 in cDenHyb (InSitus) and hybridized with samples for 3 min at 90°C, 2 min each at 80°C, 70°C, 60°C, 50°C, and 45°C, and overnight at 37°C in a humidified chamber. At 45°C, cells were washed once each with 1.5x SSC, 50% formamide / 1.5x SSC, and 1.5x SSC, then washed twice with PBS at RT. Coverslips were mounted as described above.

### EdU Pulse-Chase Analysis

EdU (5-ethynyl-2’-deoxyuridine) was diluted to 2x final concentration (60μM) in post-infection media (DMEM / 1% FBS / A-A / βME), added to an equal volume of cell media to reach a final concentration of 30μM at indicated times post-infection, and incubated for 5 min at 37°C and 5% CO_2_. For pulse only (no chase) conditions, cells were immediately processed as described above at the end of the pulse (*e*.*g*., 5 min). For pulse-chase conditions, coverslips were rinsed twice with EdU-free media at the end of the EdU pulse, then incubated in media supplemented with 30μM thymidine until the end of the experiment. The “click reaction” was performed according to the manufacturer’s protocol (Invitrogen, C10340) to conjugate a fluorescent dye-labeled (AlexaFluor 555 or AlexaFluor 647) picolyl azide to the EdU alkyne. Cells were immuno-labeled and fixed again in 3% PFA for 10 min to cross-link bound antibodies prior to the click reaction. Following the reaction, cells were washed twice with PBS and mounted as described above.

### Microscopy

Laser scanning confocal microscopy (LSCM) images were acquired on a Nikon A1R-HD laser scanning confocal microscope, using a 1.49NA 60x oil objective and 488/561/640 laser lines. Raw structured illumination images were acquired on a Nikon structured illumination microscope, using a 1.49NA 100x oil SR Apo TIRF objective, 405/488/561/647 laser lines, and an Andor iXon X3 EM-CCD 512x512 16-bit detector. Raw images were reconstructed into super-resolution (*i*.*e*., sub-diffraction limit) 3D-SIM images using the Nikon Elements software and 3D-SIM module. Reconstruction parameters for all channels and images were as follows: Illumination Modulation Contrast = 1.00, High Resolution Noise Suppression = 1.00.

Individual cells were chosen for imaging based on the presence or absence of VRCs, as marked by LT localization to bright nuclear domains. Cells with undetectable (uninfected) or diffuse (early infection) LT signal were excluded. Cells with VRCs of diverse sizes were imaged and analyzed in order to include infected cells throughout the viral replication cycle. For EdU pulse-chase experiments, cells were chosen for imaging based on the presence of VRCs and the localization of EdU signal at VRCs, which co-localizes with vDNA labeled by FISH [8]. Cells were excluded from analysis if they exhibited signs of host DNA replication, such as EdU signal along the nuclear periphery or in perinucleolar space [88–90].

### Image processing and analysis

Image processing and line scan analyses were performed using ImageJ analysis software (NIH). Reconstructed 3D-SIM images were not subjected to additional processing prior to co-localization analysis; brightness and contrast were globally adjusted in representative figure images to remove low-intensity background noise and to accurately represent the high- and low-intensity signals of interest in each channel. Co-localization analyses (Pearson’s Correlation Coefficients (PCC)) were carried out using a custom MatLab script, based on the practices outlined in [48]. The PCC measures the covariance of signal intensities between two channels and throughout the 3D volume of the image. Briefly, for each 3D image, a percentile-based threshold was applied to each fluorescent channel in the image. For each PCC analysis (*e*.*g*., of “green” signal and “red” signal), a correlation value (ranging from -1 to +1) was calculated from a paired list of voxels wherein green and red signal both exceeded their respective thresholds. PCC analysis was preferred over other methods (*e*.*g*., Mander’s Overlap Coefficients) for quantifying 3D-SIM images because it more consistently reflected changes in variable structures such as viral replication centers. Additionally, PCC analysis is sensitive to positive (attraction or interaction) and negative (repulsion or exclusion) relationships between signals.

Line scan analysis was carried out using ImageJ analysis software to illustrate spatial relationships of fluorescent signals in 3D-SIM images. For each line scan analysis, a line was drawn through an area of interest in the image (marked by a dotted white line in each supplemental figure panel) and the signal intensity of each pixel along that line was recorded for each fluorescent channel using the Plot Profile tool. The values of each signal intensity were then normalized to the minimum and maximum values of each channel to yield a common value scale from 0–1.

Dim and focal RPA32 subsets were isolated for analysis in several experiments (Figs 1, 2, 4 and 6; S1 Fig). ImageJ analysis software was used to visualize and identify threshold values. Briefly, several threshold values were tested to optimize the isolation of dim focal RPA32 signals from background noise. These thresholds were based on percentiles of signal intensity for the entire z-stack of individual images, as shown in S1C Fig. For example, only the brightest 1% of voxels exceed a 99.0% threshold. Several thresholds were tested against a series of images, wherein voxel values exceeding a 99.95% threshold reliably isolated focal RPA32 voxels, voxel values in the 99.75–99.94% range isolated dim RPA32 voxels, and the remaining voxels (0–99.74%) were discarded as background noise or non-VRC signal (S1D Fig). These threshold values were validated against images in each relevant experiment, and they were held constant across all images and between experiments. Once isolated, no further processing of dim and focal regions was carried out (*e*.*g*., volumetric or morphological thresholds); the threshold values were entered for PCC analysis, as described above. Intensity threshold values of 99.75% was determined and validated similarly for other fluorescent channels (*e*.*g*., LT).

### Statistical analysis

All error bars represent standard deviation. Statistical significance was calculated using either paired or unpaired two-tailed Student’s t-tests [91]. Paired t-tests were used to assess the mean PCC values of different combinations of fluorescent channels within each image (*e*.*g*., LT with dim RPA32 or with focal RPA32 in Fig 1C). Unpaired t-tests were used to assess the mean PCC values of different experimental conditions (*e*.*g*., EdU and LT after increasing chase durations in Fig 3D). Welch’s correction was applied to all unpaired t-tests to account for the possibility of unequal variance or sample size between conditions. These analyses were carried out in GraphPad Prism software (version 8.3.0) and confirmed in Microsoft Excel. p values <0.05 are indicated with *, p values <0.01 are indicated with **, p values <0.001 are indicated with ***, and non-significant differences are marked “ns.” The reported n values represent individual nuclei from one biological replicate.

## Supporting information

S1 Fig MuPyV replication centers are organized into subdomains. **(A)** Threshold segmentation of single z-planes in RPA32 (left) and LT (right) channels identified regions of dense (bright) fluorescence signal, which were outlined in yellow and overlaid on the other channels to highlight juxtaposition of VRC subdomains. **(B)** Line scan analysis of VRCs imaged by laser scanning confocal microscopy (LSCM) or 3D structured illumination microscopy (3D-SIM). Fluorescence intensities along dotted white lines were analyzed for each fluorescent channel and normalized to min and max values within each channel (RPA32 = green, LT = red, vDNA(FISH) = gray). **(C)** Example of percentile-based segmentations of RPA32 signal, based on entire 3D-SIM image stack (~9.44x10^6^ voxels) but displayed on a single z-plane. Voxel values exceeding indicated thresholds are displayed in white. Dotted white line indicates nuclear border; dotted white box indicates highlighted VRC. **(D)** Example of non-overlapping percentile-based threshold ranges to segment background (black), non-focal dim (blue), and focal bright (red) signal in the RPA32 channel. Percentile ranges are listed for each pool. Scale bars: Nucleus = 5μm, Crop = 0.5μm. n = 13 nuclei.
(TIF)

S2 Fig Focal RPA32 is associated with DDR signaling proteins. **(A)** Line scan analysis of pRPA32^S4S8^ localization. Fluorescence intensities along dotted white line were analyzed for each fluorescent channel and normalized to min and max values within each channel (RPA32 = green, LT = red, pRPA32^S4S8^ = gray). **(B)** Line scan analysis of pATM^S1981^ localization. Fluorescence intensities along dotted white line were analyzed for each fluorescent channel and normalized to min and max values within each channel (RPA32 = green, LT = red, pATM^S1981^ = gray). Scale Bars = 0.5μm.
(TIF)

S3 Fig Nascent MuPyV DNA rapidly dissociates from LT. **(A-B)** Line scan analysis of EdU-labeled vDNA (gray) and LT (red) localization at selected time points. Fluorescence intensities along dotted white lines were analyzed for each fluorescent channel and normalized to min and max values within each channel. Scale Bar = 0.5μm.
(TIF)

S4 Fig MuPyV DNA progresses from LT to focal RPA32 subdomains. Line scan analysis of vDNA (EdU) localization relative to LT and RPA32. Fluorescence intensities along dotted white lines were analyzed for each fluorescent channel and normalized to min and max values within each channel (RPA32 = green, LT = red, vDNA (EdU) = gray). Scale bars = 0.5μm.
(TIF)

S5 Fig ST contributes to vDNA relocalization after synthesis. Line scan analysis of **(A)** 808A-infected or **(B)** NG18-infected cells to highlight vDNA (EdU) localization relative to LT and RPA32. Fluorescence intensities along dotted lines were analyzed for each fluorescent channel and normalized to min and max values within each channel (RPA32 = green, LT = red, vDNA (EdU) = gray). Scale bars = 0.5μm. **(C)** PCC values of LT (>99.75%) with dim RPA32 (99.75–99.94%) or focal RPA32 (>99.95%) (n = 24 nuclei per virus). Unpaired t-tests were used to compare mean values (*** = p<0.001; ** = p<0.01; ns = p>0.05).
(TIF)

S6 Fig pRPA32^S4S8^ and pATM^1981^ localization is disrupted in NG18 VRCs. **(A)** Line scan analysis of pRPA32^S4S8^ localization in an NG18-infected cell. Fluorescence intensities along dotted white line were analyzed for each fluorescent channel and normalized to min and max values within each channel (RPA32 = green, LT = red, pRPA32^S4S8^ = gray). **(B)** Line scan analysis of pATM^S1981^ localization in an NG18-infected cell. Fluorescence intensities along dotted while line were analyzed for each fluorescent channel and normalized to min and max values within each channel (RPA32 = green, LT = red, pATM^S1981^ = gray). Scale Bars = 0.5μm.
(TIF)

S7 Fig Hydroxyurea disrupts VRC organization and vDNA relocalization. **(A-C)** Line scan analysis of vDNA (EdU) localization relative to LT and RPA32 in the presence or absence of hydroxyurea (HU). Fluorescence intensities along dotted white lines were analyzed for each fluorescent channel and normalized to min and max values within each channel (RPA32 = green, LT = red, vDNA(EdU) = gray). Scale Bar = 0.5μm.
(TIF)

S8 Fig VRC disruption by hydroxyurea is reversible. **(A-C)** Line scan analysis of vDNA (FISH) localization relative to LT and RPA32 at different times after release from HU. Fluorescence intensities along dotted white lines were analyzed for each fluorescent channel and normalized to min and max values within each channel (RPA32 = green, LT = red, vDNA(FISH) = gray). Scale Bar = 0.5μm.
(TIF)
