# Supplementary figures and images for: Murine polyomavirus DNA transitions through spatially distinct nuclear replication subdomains during infection
Source: PLoS Pathog. 2020 Mar 23;16(3):e1008403. doi: 10.1371/journal.ppat.1008403 (PMC7117779; doi:10.1371/journal.ppat.1008403)

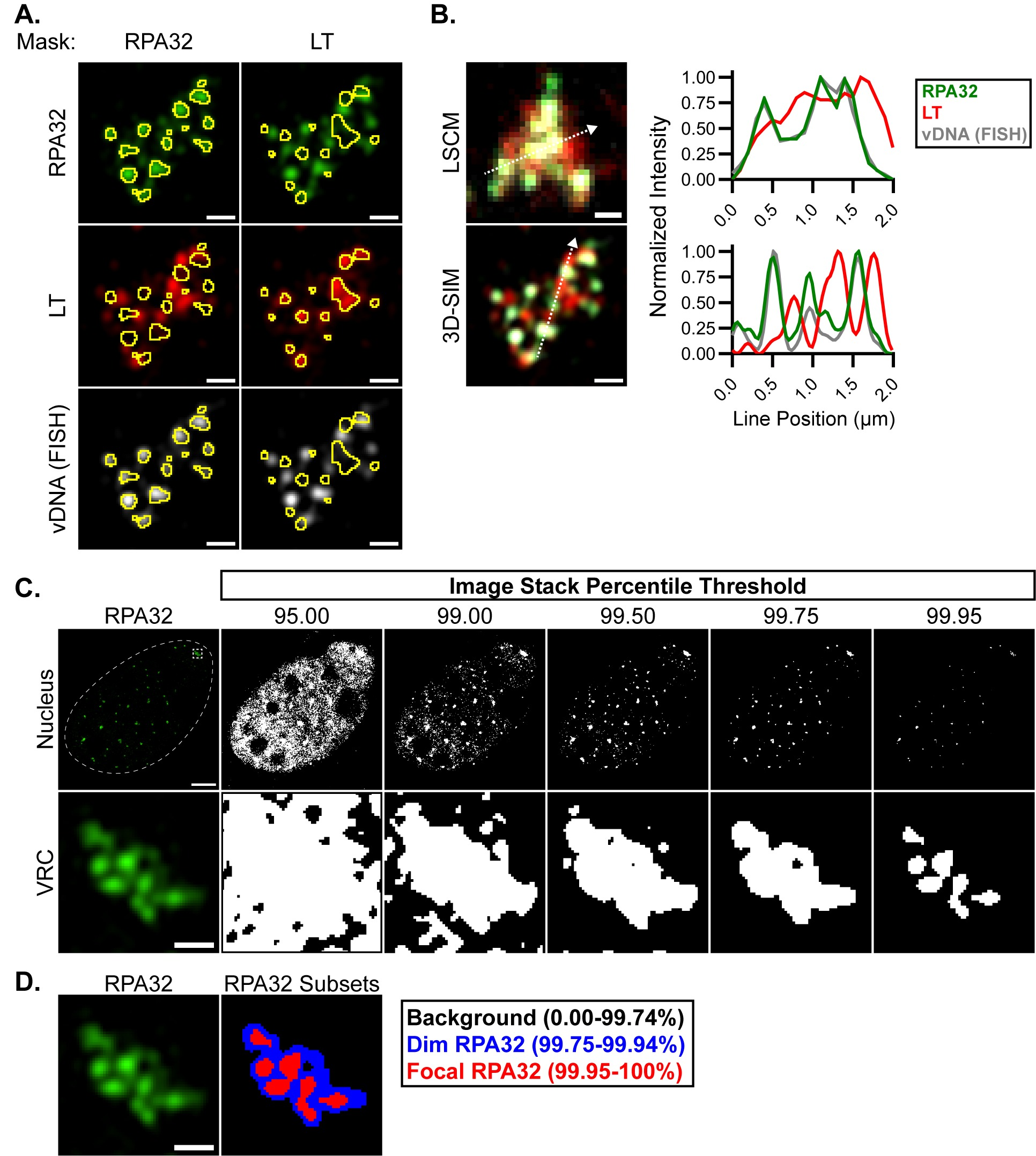

Supplement: S1 Fig — (A) Threshold segmentation of single z-planes in RPA32 (left) and LT (right) channels identified regions of dense (bright) fluorescence signal, which were outlined in yellow and overlaid on the other channels to highlight juxtaposition of VRC subdomains. (B) Line scan analysis of VRCs imaged by laser scanning confocal microscopy (LSCM) or 3D structured illumination microscopy (3D-SIM). Fluorescence intensities along dotted white lines were analyzed for each fluorescent channel and normalized to min and max values within each channel (RPA32 = green, LT = red, vDNA(FISH) = gray). (C) Example of percentile-based segmentations of RPA32 signal, based on entire 3D-SIM image stack (~9.44x10^6^ voxels) but displayed on a single z-plane. Voxel values exceeding indicated thresholds are displayed in white. Dotted white line indicates nuclear border; dotted white box indicates highlighted VRC. (D) Example of non-overlapping percentile-based threshold ranges to segment background (black), non-focal dim (blue), and focal bright (red) signal in the RPA32 channel. Percentile ranges are listed for each pool. Scale bars: Nucleus = 5μm, Crop = 0.5μm. n = 13 nuclei. (TIF) [file ppat.1008403.s001.tif]

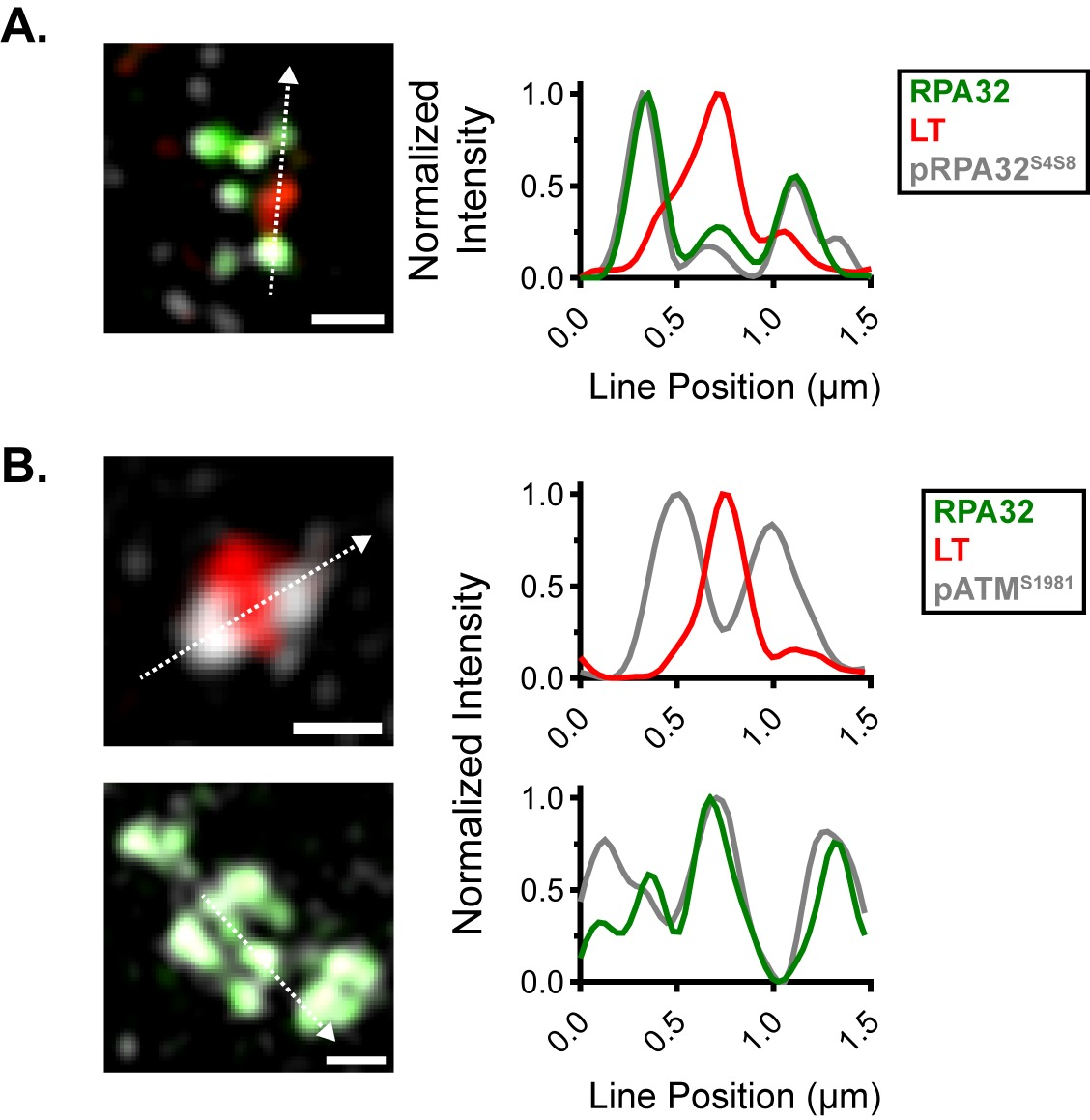

Supplement: S2 Fig — (A) Line scan analysis of pRPA32S4S8 localization. Fluorescence intensities along dotted white line were analyzed for each fluorescent channel and normalized to min and max values within each channel (RPA32 = green, LT = red, pRPA32S4S8 = gray). (B) Line scan analysis of pATMS1981 localization. Fluorescence intensities along dotted white line were analyzed for each fluorescent channel and normalized to min and max values within each channel (RPA32 = green, LT = red, pATMS1981 = gray). Scale Bars = 0.5μm. (TIF) [file ppat.1008403.s002.tif]

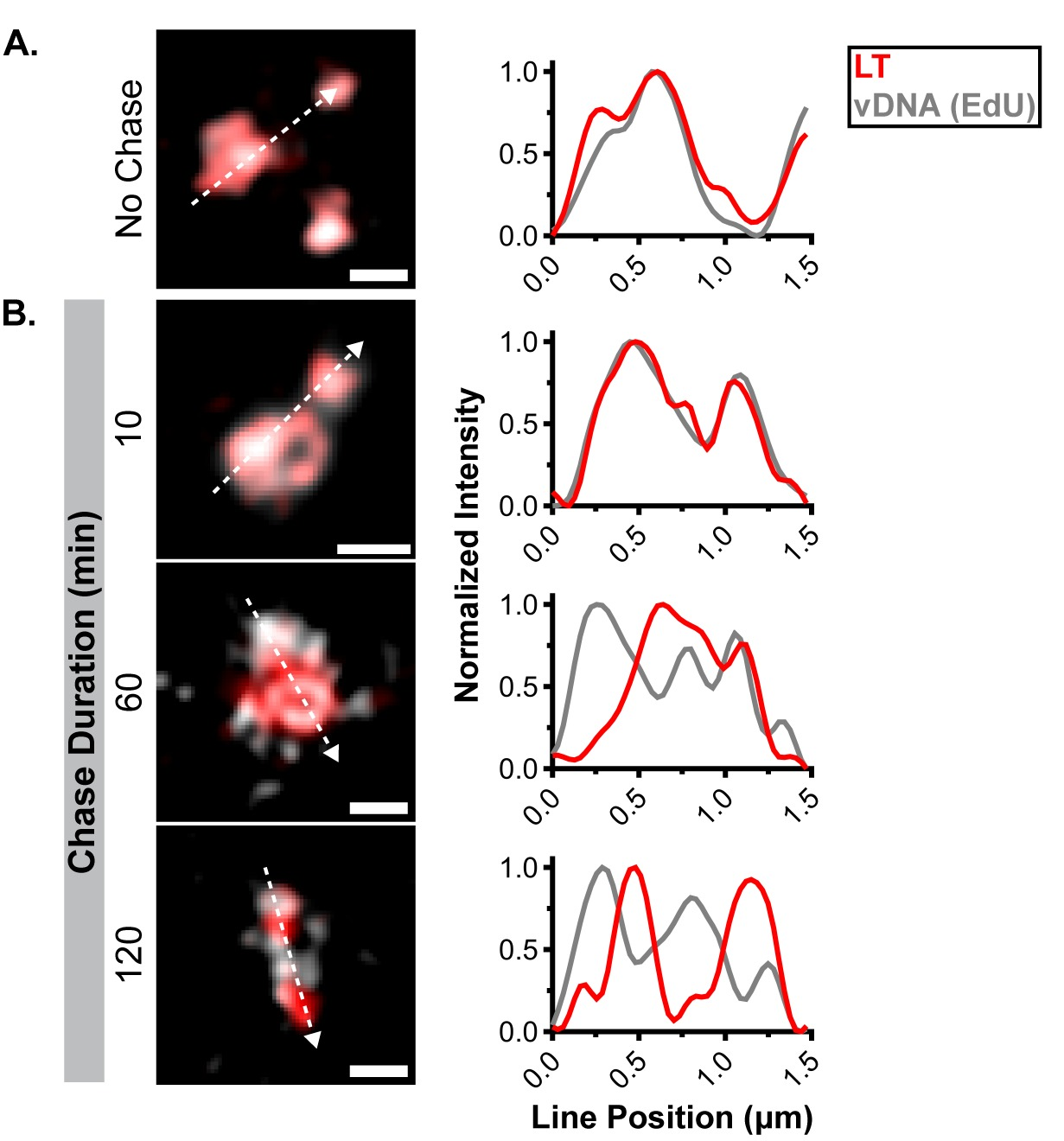

Supplement: S3 Fig — (A-B) Line scan analysis of EdU-labeled vDNA (gray) and LT (red) localization at selected time points. Fluorescence intensities along dotted white lines were analyzed for each fluorescent channel and normalized to min and max values within each channel. Scale Bar = 0.5μm. (TIF) [file ppat.1008403.s003.tif]

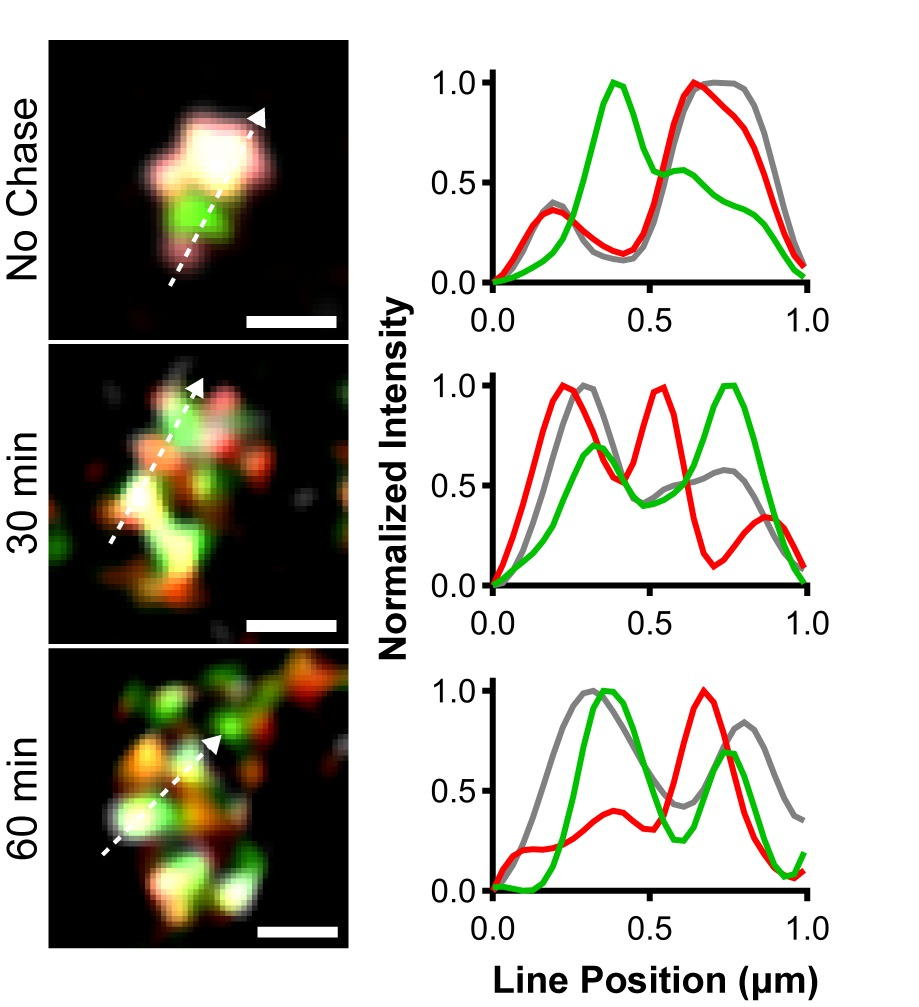

Supplement: S4 Fig — Line scan analysis of vDNA (EdU) localization relative to LT and RPA32. Fluorescence intensities along dotted white lines were analyzed for each fluorescent channel and normalized to min and max values within each channel (RPA32 = green, LT = red, vDNA (EdU) = gray). Scale bars = 0.5μm. (TIF) [file ppat.1008403.s004.tif]

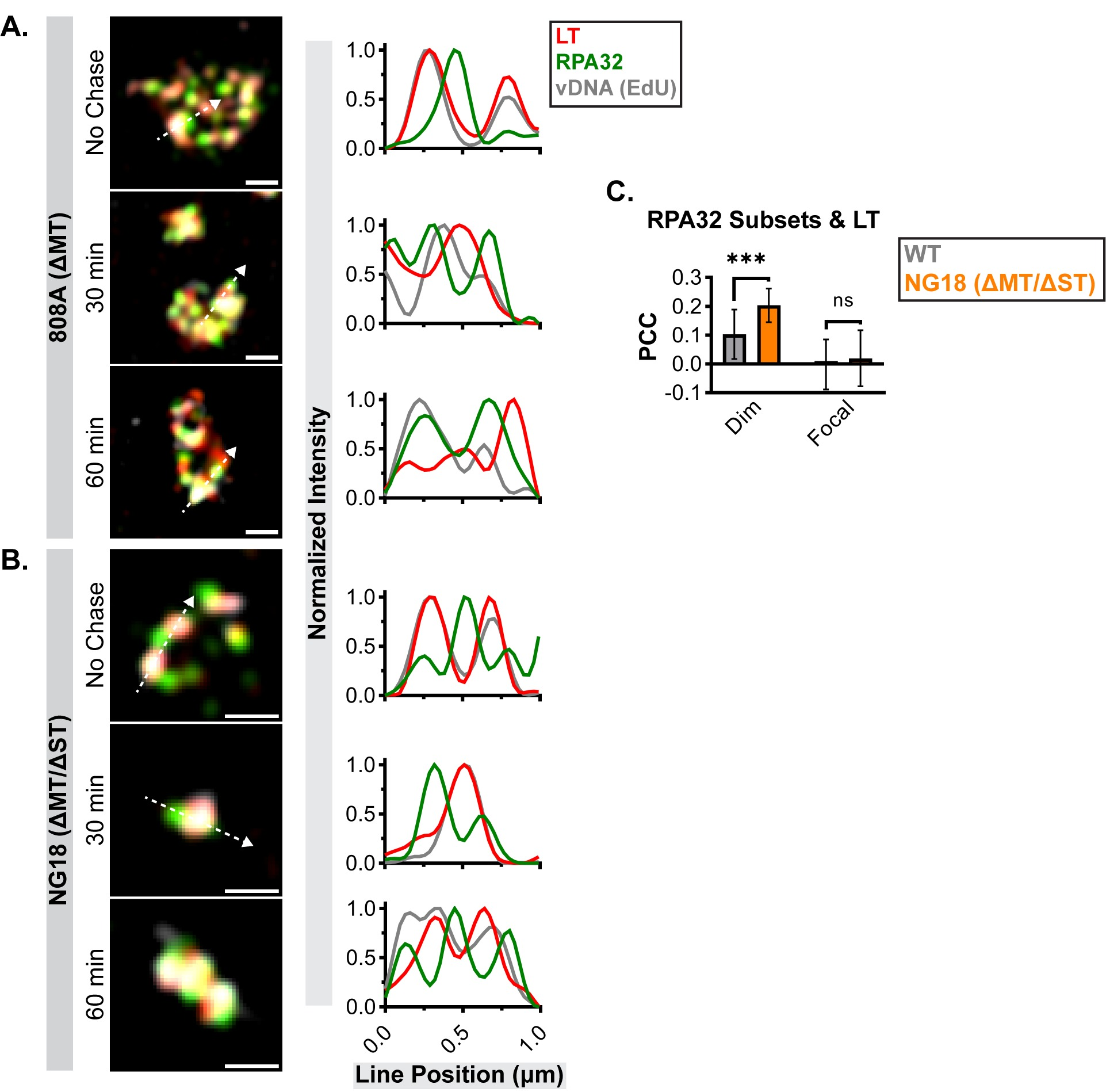

Supplement: S5 Fig — Line scan analysis of (A) 808A-infected or (B) NG18-infected cells to highlight vDNA (EdU) localization relative to LT and RPA32. Fluorescence intensities along dotted lines were analyzed for each fluorescent channel and normalized to min and max values within each channel (RPA32 = green, LT = red, vDNA (EdU) = gray). Scale bars = 0.5μm. (C) PCC values of LT (>99.75%) with dim RPA32 (99.75–99.94%) or focal RPA32 (>99.95%) (n = 24 nuclei per virus). Unpaired t-tests were used to compare mean values (*** = p<0.001; ** = p<0.01; ns = p>0.05). (TIF) [file ppat.1008403.s005.tif]

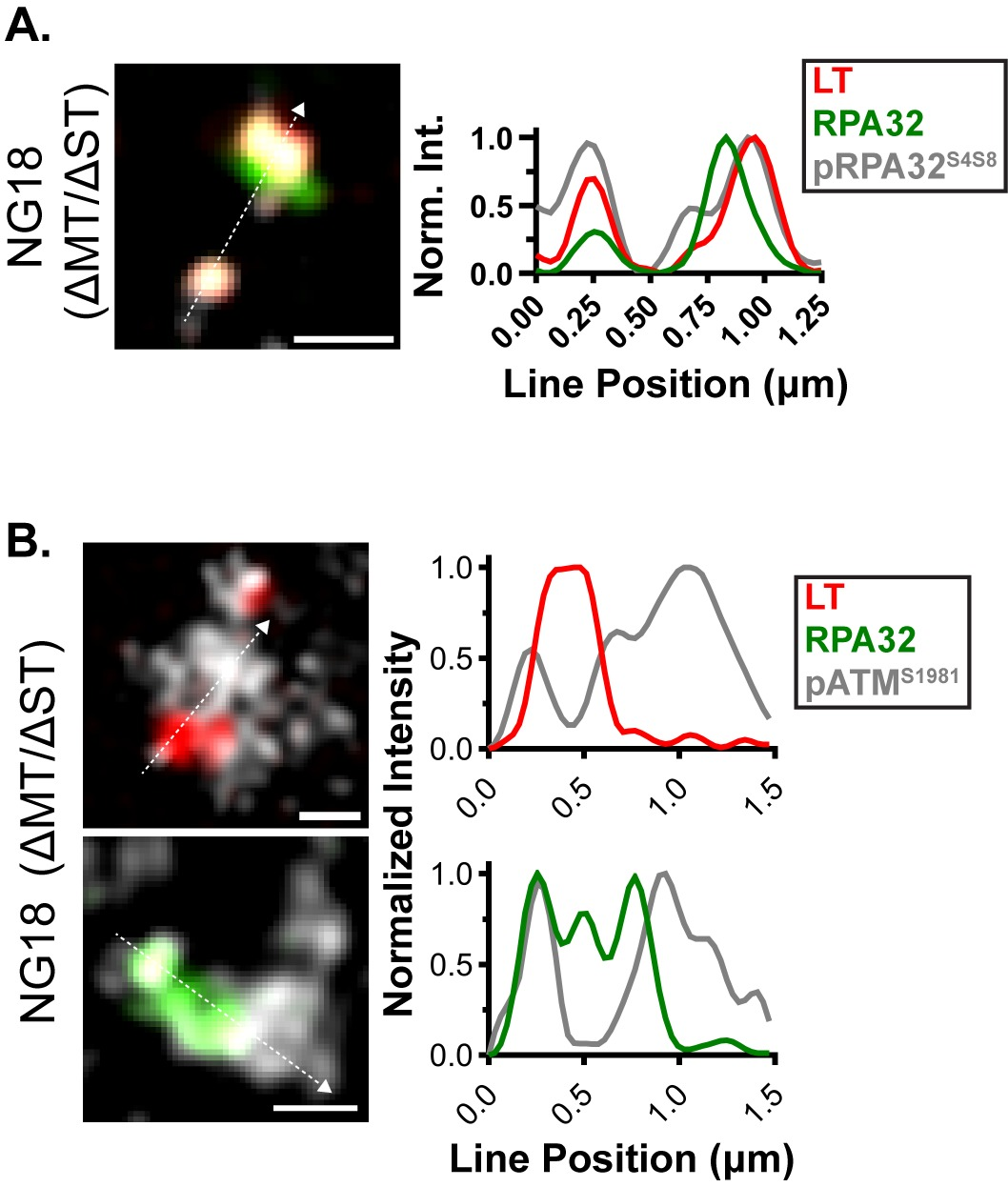

Supplement: S6 Fig — (A) Line scan analysis of pRPA32S4S8 localization in an NG18-infected cell. Fluorescence intensities along dotted white line were analyzed for each fluorescent channel and normalized to min and max values within each channel (RPA32 = green, LT = red, pRPA32S4S8 = gray). (B) Line scan analysis of pATMS1981 localization in an NG18-infected cell. Fluorescence intensities along dotted while line were analyzed for each fluorescent channel and normalized to min and max values within each channel (RPA32 = green, LT = red, pATMS1981 = gray). Scale Bars = 0.5μm. (TIF) [file ppat.1008403.s006.tif]

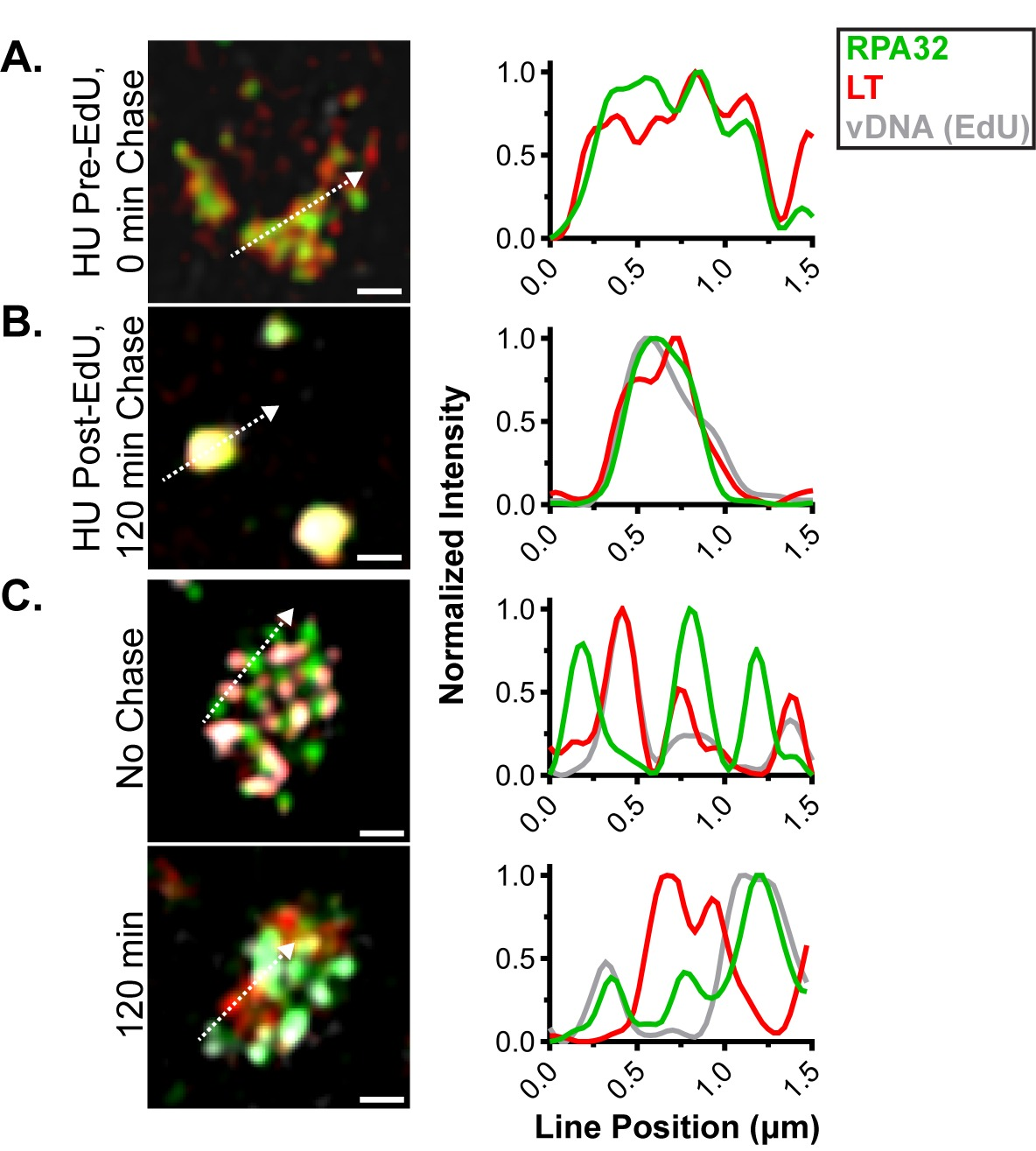

Supplement: S7 Fig — (A-C) Line scan analysis of vDNA (EdU) localization relative to LT and RPA32 in the presence or absence of hydroxyurea (HU). Fluorescence intensities along dotted white lines were analyzed for each fluorescent channel and normalized to min and max values within each channel (RPA32 = green, LT = red, vDNA(EdU) = gray). Scale Bar = 0.5μm. (TIF) [file ppat.1008403.s007.tif]

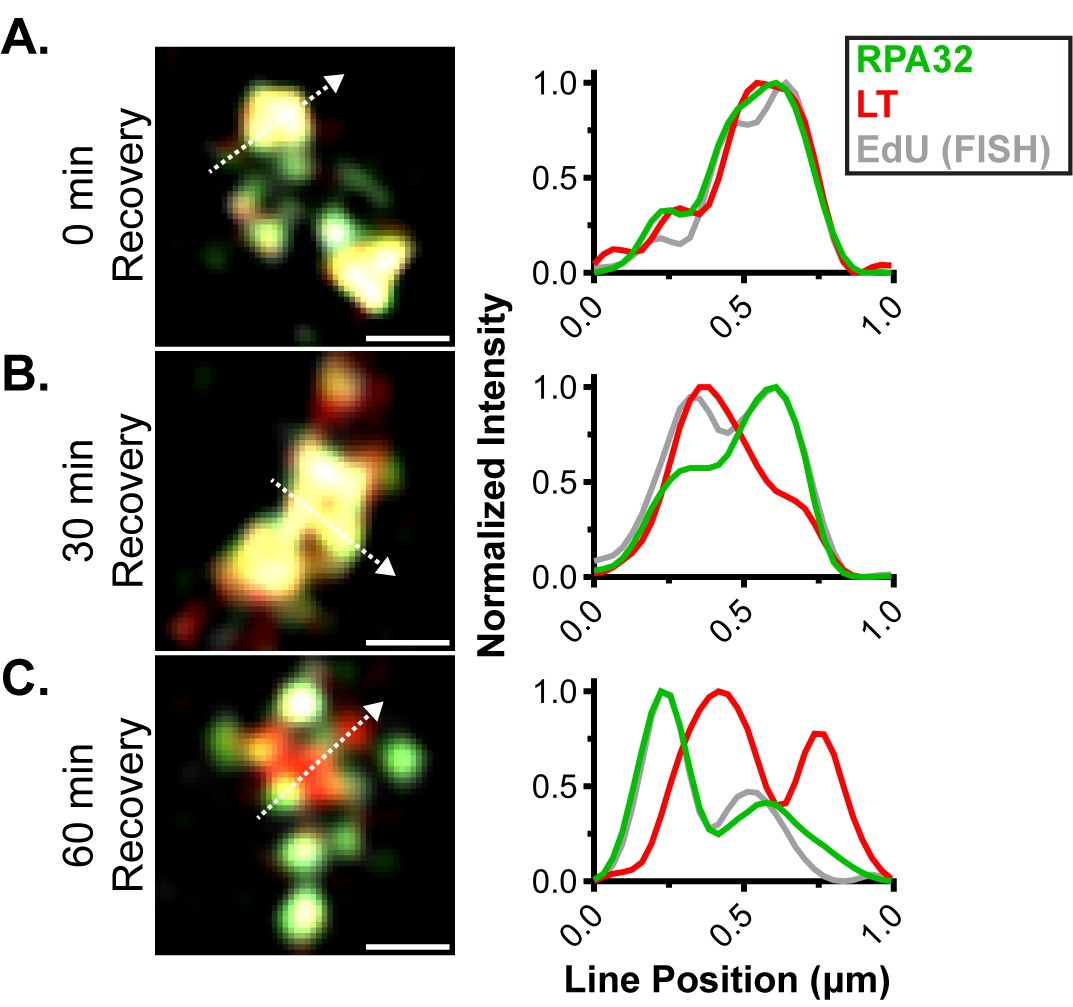

Supplement: S8 Fig — (A-C) Line scan analysis of vDNA (FISH) localization relative to LT and RPA32 at different times after release from HU. Fluorescence intensities along dotted white lines were analyzed for each fluorescent channel and normalized to min and max values within each channel (RPA32 = green, LT = red, vDNA(FISH) = gray). Scale Bar = 0.5μm. (TIF) [file ppat.1008403.s008.tif]
